# Finite Element Model Updating for Rotating Machinery: Methods, Applications, and Future Directions—A Review

**DOI:** 10.3390/s26185824

**Published:** 2026-09-14

**Authors:** Donghee Park, Jongyoung Moon, Jaegwang Yoon, Byeong Keun Choi

**Affiliations:** 1 DAVISS Inc., Jinju 52828, Republic of Korea; pdh@gnu.ac.kr; 2 Department of Energy and Mechanical Engineering, Gyeongsang National University, Tongyeong 53064, Republic of Korea; mjy8415@gnu.ac.kr (J.M.); yjg298@gnu.ac.kr (J.Y.)

**Keywords:** finite element model updating, rotating machinery, rotor dynamics, parameter identification, uncertainty quantification, digital twin

## Abstract

**Highlights:**

**What are the main findings?**

- FEMU is most mature for rotor–bearing calibration, bearing/support identification, and operational-response-based parameter updating.
- Experimental validation remains limited for nonlinear bearing behavior, rotor–stator contact, and compound-fault parameter estimation.

**What are the implications of the main findings?**

- Future FEMU should emphasize parameter identifiability, uncertainty quantification, and multi-speed or multi-response validation.
- FEMU can support lifecycle digital twins through physics-based model synchronization combined with validated surrogate or AI-assisted estimation.

**Abstract:**

Finite element model updating (FEMU) provides a physics-based approach for reducing discrepancies between numerical models and measured responses by estimating uncertain physical parameters. Its application to rotating machinery is challenging because rotor dynamics are strongly affected by rotational speed, bearing and support properties, nonlinear interactions, operating conditions, measurement limitations, and parameter correlation. This review examines FEMU methods and applications for rotating machinery, distinguishing direct FEMU studies from nonlinear, uncertainty, surrogate, and AI-based studies that primarily provide enabling techniques. Direct matrix correction, sensitivity-based, optimization-based, surrogate-assisted, Bayesian, AI-driven, and hybrid approaches are compared in terms of physical interpretability, identifiability, computational demand, uncertainty treatment, and validation. The reviewed literature indicates that FEMU is most mature for rotor–bearing calibration, bearing and support parameter identification, and operational-response-based updating, whereas experimentally validated inverse estimation of nonlinear and compound-fault parameters remains limited. Based on these findings, a lifecycle-oriented FEMU framework and research roadmap are proposed, emphasizing multi-condition identifiability, uncertainty-aware updating, computational efficiency, independent validation, and governed synchronization. Surrogate and AI-assisted estimation should remain connected to validated high-fidelity physical models and defined operating domains for credible condition assessment and digital-twin applications.

## 1. Introduction

Rotating machinery, including turbines, compressors, pumps, generators, motors, and transmission systems, plays a central role in power generation, manufacturing, transportation, and other industrial sectors. These machines commonly operate under high rotational speeds and variable loads, and their dynamic behavior directly affects operational reliability, safety, and maintenance cost. Mechanical faults such as unbalance, misalignment, bearing degradation, shaft cracks, looseness, and rotor–stator rubbing produce measurable changes in vibration responses and may cause severe secondary damage if they are not detected at an early stage. Consequently, vibration-based condition monitoring has become an essential component of predictive-maintenance strategies for rotating machinery [1,2,3].

Finite element models are widely used to predict the dynamic characteristics of rotating systems, including natural frequencies, mode shapes, critical speeds, unbalance responses, and stability boundaries. However, numerical models inevitably differ from their physical counterparts because of geometric simplification, nominal material properties, idealized interfaces, uncertain support conditions, and simplified representations of bearings, couplings, and foundations. These discrepancies can reduce the reliability of predicted modal and operational responses, particularly when flexible supports, speed-dependent effects, or nonlinear behavior are involved [4,5,6].

Finite element model updating (FEMU) reduces model–measurement discrepancies by estimating uncertain model parameters from experimentally measured responses. Typical updating parameters include material properties, joint and support stiffness, bearing coefficients, damping characteristics, mass distribution, and boundary conditions. The updated model is expected not only to reproduce the measurements used for calibration but also to provide reliable predictions under operating conditions that were not included in the updating process [4,5,6,7,8,9,10,11,12].

The application of FEMU to rotating machinery presents requirements that differ from those of stationary structural systems. The dynamic response of a rotor varies with rotational speed, bearing condition, support characteristics, thermal state, and fault severity. Gyroscopic effects, fluid-film forces, rotor–stator interaction, and coupled faults may also introduce speed-dependent or nonlinear responses. Therefore, a model calibrated at a single operating condition may not remain valid over the complete operating range. Parameter selection, measurement selection, model-form adequacy, and validation across multiple operating conditions are consequently central issues in rotating-machinery FEMU [13,14,15,16,17,18].

Measurement noise, limited sensor locations, incomplete modal information, and parameter correlation further complicate the updating problem. Different combinations of structural, bearing, and support parameters may produce similar measured responses, resulting in non-unique or poorly identifiable solutions. Uncertainty-aware methods and computationally efficient surrogate models have therefore become increasingly relevant when FEMU is used for predictive or maintenance-related decisions [10,11,12,13,14,15].

Recent developments in surrogate modeling and artificial intelligence have extended FEMU beyond conventional offline optimization. Data-driven models can accelerate repeated finite element evaluations or approximate the inverse relationship between measured responses and uncertain physical parameters. However, their reliability remains dependent on the coverage and physical fidelity of the training data, particularly when simulation-generated data are used. AI-assisted FEMU should therefore preserve physical parameter constraints and be validated using the original numerical model and independent experimental measurements [19,20,21,22,23,24,25,26,27,28].

FEMU can also support condition monitoring, prognostics, and digital-twin applications. Model–measurement residuals and updated physical parameters can provide information on changes in machinery condition, while continuously or periodically updated models can improve fault-severity estimation and future-response prediction. Within a digital-twin framework, FEMU acts as a synchronization mechanism between the physical machine and its virtual representation. Nevertheless, practical implementation requires reliable update criteria, uncertainty assessment, computational efficiency, and independent validation [29,30,31,32,33].

Previous reviews have examined structural FEMU, uncertainty quantification, rotor dynamics, vibration-based diagnosis, prognostics, and digital-twin technologies as largely separate research areas. Classical FEMU reviews primarily focus on numerical formulations and updating algorithms, whereas rotating-machinery diagnostic reviews generally emphasize signal processing and data-driven classification. Digital-twin reviews address virtual–physical integration but often provide limited discussion of the rotor-specific dynamic characteristics that affect model-updating reliability. Unlike previous reviews that primarily address FEMU methodology, rotor uncertainty and diagnosis, or digital-twin technologies separately, the present review evaluates these developments within a common rotating-machinery FEMU framework. In particular, it distinguishes demonstrated physical-parameter updating from FEMU-enabling studies and compares them in terms of parameter identifiability, validation evidence, uncertainty treatment, and applicability to lifecycle-oriented digital twins [4,6,15,20,29,30,31,32,33].

Accordingly, this review examines the development and application of FEMU for rotating machinery. Its principal contributions are as follows:

1. FEMU methods are classified and compared according to their physical interpretability, computational requirements, uncertainty treatment, and suitability for rotating systems.
2. Rotating-machinery-specific sources of model–measurement discrepancy are related to updating-parameter selection, identifiability, and validation requirements.
3. Direct rotating-machinery FEMU studies are distinguished from nonlinear modeling, AI, and uncertainty studies that provide enabling methods but do not directly perform experimental model updating.
4. The potential roles of FEMU in condition monitoring, prognostics, and digital-twin synchronization are evaluated, and the major requirements for reliable lifecycle-oriented implementation are identified.

**Figure 1:**
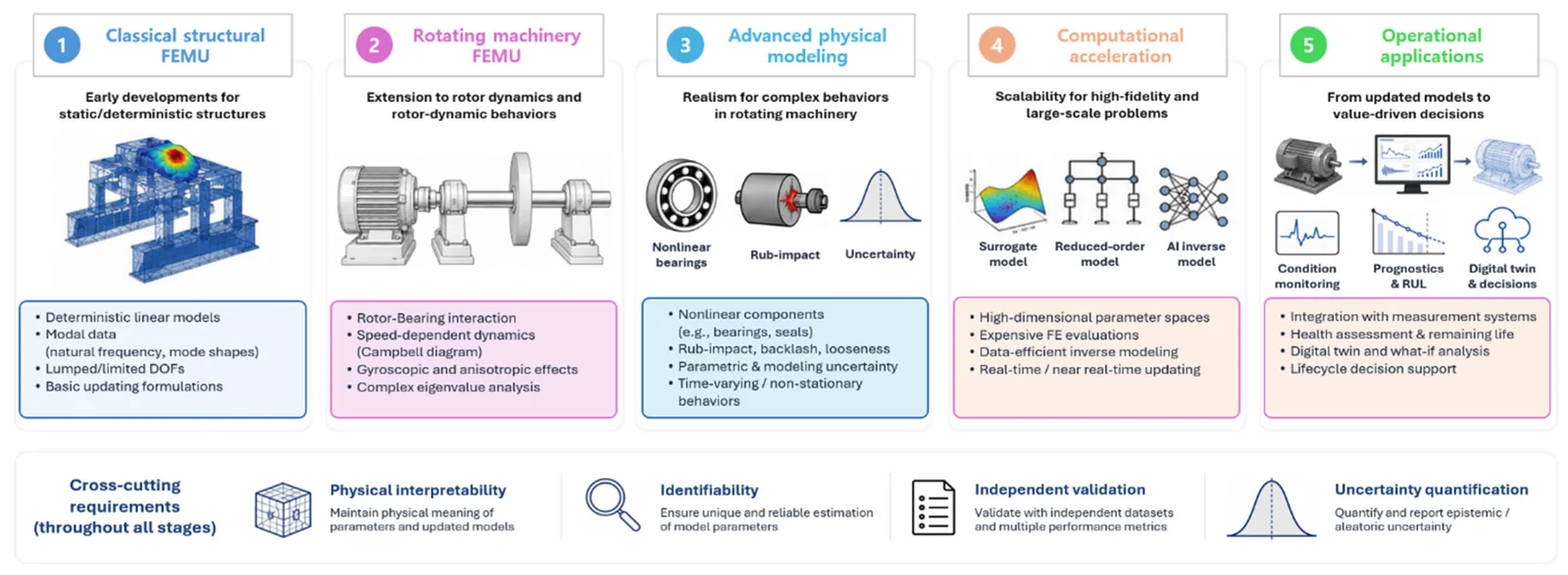
Evolution and scope of finite element model updating for rotating machinery.

Figure 1 summarizes the scope of this review, connecting classical structural FEMU with rotating-system applications, nonlinear and uncertainty-aware modeling, computational acceleration, condition monitoring, prognostics, and digital twins. Physical interpretability, parameter identifiability, uncertainty quantification, and independent validation are treated as common requirements across these research areas.

## 2. Review Scope and Literature Classification

### 2.1. Scope of the Review

This review focuses on finite element model updating (FEMU) methods and their application to rotating machinery. The primary scope covers rotor–bearing systems, shafts, disks, couplings, supports, wind turbine blades, and multi-component rotating systems. Particular attention is given to characteristics that distinguish rotating machinery from stationary structures, including speed-dependent dynamics, gyroscopic effects, bearing and support properties, nonlinear forces, uncertain operating conditions, and limited measurement availability.

The reviewed literature includes both direct FEMU studies and enabling studies. Direct FEMU studies are defined here as investigations in which measured responses are used to estimate or correct physical parameters of a numerical rotating-system model. Representative targets include shaft and support properties, bearing stiffness and damping, material parameters, boundary conditions, and fault-related variables.

Enabling studies are included when they provide physical models, computational techniques, or data-processing methods that can be incorporated into FEMU. These studies include nonlinear rotor-dynamic modeling, uncertainty quantification, surrogate modeling, inverse learning, synthetic-data generation, prognostics, and digital-twin synchronization [13,14,15,16,17,18,19,20,21,22,23,24,25,26,27,28,29,30,31,32,33]. They are distinguished from direct FEMU applications throughout the review to avoid treating all simulation- or AI-based rotating-machinery studies as model-updating research.

Studies based exclusively on data-driven fault classification are outside the principal scope when they do not contain a physical model, parameter-estimation procedure, simulation-based interpretation, or virtual–physical synchronization mechanism. Such studies are discussed only when their methods contribute directly to hybrid FEMU, simulation-to-real transfer, synthetic-data generation, or physics-based diagnosis.

### 2.2. Literature Collection and Selection Principles

Relevant literature was identified through the Web of Science Core Collection and Scopus, supplemented by Google Scholar and the reference lists of established publications on finite element model updating (FEMU) and rotor dynamics. The final database search was conducted on 4 September 2026. The database search was restricted to English-language articles and review articles, while no publication-year restriction was applied because foundational studies were required to establish the theoretical development of FEMU and rotating-system dynamics [4,5,6,7,10,11,12,18].

The primary database search query was (“finite element model updating” OR “model updating” OR “model calibration” OR “parameter identification”) AND (“rotor” OR “rotor-bearing” OR “shaft” OR “journal bearing” OR “rotating machinery”). In the Web of Science Core Collection, the query was applied to the Topic field, whereas in Scopus it was applied to the Article title, Abstract, and Keywords fields. Supplementary searches used combinations of FEMU and rotating-machinery terms with topics relevant to the extension of rotating-machinery FEMU, including “nonlinear dynamics,” “uncertainty,” “surrogate model,” “artificial intelligence,” “condition monitoring,” “prognostics,” and “digital twin” [9,12,15,19,29,30,31,32,33].

The literature base primarily included peer-reviewed journal articles, foundational academic books, major review papers, and the reference lists of relevant FEMU and rotor-dynamic studies. Studies were considered when they addressed one or more of the following topics: structural FEMU and physical-parameter identification [4,5,6,7,8,9,10,11,12]; rotor and rotor–bearing model calibration and bearing-parameter identification [13,14]; speed-dependent and nonlinear rotor dynamics [15,16,17,18]; deterministic and probabilistic uncertainty treatment [10,11,12,15,17]; surrogate-assisted and AI-based inverse modeling [9,19,20,21,22,23,24,25,26,27,28]; FEMU-supported condition monitoring and prognostics [20,21,22,23,24,25,26,27,28,29,30,31]; and digital-twin frameworks involving physical-model synchronization [29,30,31,32,33].

Direct FEMU studies were distinguished from FEMU-enabling studies according to the role of experimental measurements and physical-model parameter estimation. A study was classified as direct FEMU when measured responses were explicitly used to estimate or correct physical parameters of a numerical physical model, as demonstrated in representative rotor, bearing, and rotating-structure applications [13,14]. Studies that provided nonlinear physical models, uncertainty-analysis methods, surrogate models, AI-based inverse techniques, synthetic-data approaches, prognostic methodologies, or digital-twin frameworks without completing experimental physical-parameter updating were classified as FEMU-enabling studies [15,16,17,18,19,20,21,22,23,24,25,26,27,28,29,30,31,32,33]. Studies based exclusively on data-driven fault classification without physical-model calibration, parameter identification, simulation-based physical interpretation, or virtual–physical synchronization were excluded from the principal FEMU evidence base.

Foundational publications were retained when they established the theoretical basis of FEMU, including direct correction, sensitivity analysis, objective-function formulation, regularization, parameter identifiability, and uncertainty treatment [4,5,6,7,8,9,10,11,12]. More recent publications were included when they extended these foundations to rotating systems, nonlinear behavior, computationally efficient updating, AI-assisted inverse analysis, condition assessment, prognostics, or digital-twin implementation [13,14,15,16,17,18,19,20,21,22,23,24,25,26,27,28,29,30,31,32,33]. Duplicate and bibliographically unverifiable publications were excluded. When both preprint and peer-reviewed versions were available, the peer-reviewed journal publication was preferentially retained. Bibliographic information, including authorship, publication year, journal, volume, page or article number, and DOI, was checked before inclusion.

Under the predefined search conditions, the two database searches returned 1380 database records before cross-database deduplication, comprising 585 records from the Web of Science Core Collection and 795 records from Scopus. The retrieved literature was screened for relevance to the scope of this review based on titles and abstracts, with full texts examined where necessary. Studies were excluded from the principal evidence base when they were unrelated to rotating machinery, did not involve a physics-based or finite element model, or did not provide either direct physical-parameter updating or a clearly relevant FEMU-enabling methodology. Following this selection process, 18 representative studies were retained for detailed case-based comparison of direct FEMU and FEMU-enabling applications. Additional primary studies, review articles, and foundational references were retained to support the methodological, theoretical, and contextual discussions throughout the review.

Because the reviewed studies differ substantially in rotating-system configuration, numerical-model fidelity, measurement type, updating parameters, operating conditions, validation procedures, and reported performance metrics, a statistical meta-analysis based on the reported numerical performance was not considered appropriate. Direct numerical ranking across these studies may be misleading because differences in residual error or estimation accuracy can arise from differences in system complexity, parameter dimensionality, measurement quality, operating range, and validation conditions rather than from the updating algorithm itself. Therefore, this review adopts a structured comparative synthesis based on common criteria, including physical interpretability, parameter identifiability, uncertainty treatment, validation level, computational requirement, and principal limitations. The absence of a common benchmark for direct quantitative comparison is also identified as an important research gap and is further addressed in Section 6.5.5.

### 2.3. Classification of the Reviewed Literature

The reviewed literature was organized into five categories according to its role in rotating-machinery FEMU.

The first category comprises the fundamental FEMU methods, including direct matrix correction, sensitivity-based updating, optimization, surrogate-assisted estimation, Bayesian inference, and uncertainty-aware approaches [4,5,6,7,8,9,10,11,12]. These studies establish the mathematical and computational basis for parameter identification.

The second category contains direct applications to rotating machinery, in which experimental modal or operational responses are used to update rotor, bearing, support, or structural parameters [13,14]. These studies constitute the core evidence for the practical applicability of rotating-system FEMU.

The third category includes nonlinear and uncertainty-related rotor-dynamic studies. These investigations address fluid-film instability, clearance, friction, rotor–stator contact, rub-impact, misalignment, compound faults, and uncertain system parameters [15,16,17,18]. When experimental parameter updating is not performed, these studies are treated as sources of physical models, response characteristics, and candidate updating parameters rather than as direct FEMU applications.

The fourth category covers surrogate, AI, and hybrid approaches that accelerate repeated numerical analyses or approximate the forward and inverse relationships between physical parameters and measured responses [19,20,21,22,23,24,25,26,27,28]. Their relevance is assessed according to physical interpretability, training-domain dependence, computational efficiency, and compatibility with finite element validation.

The fifth category includes condition monitoring, prognostics, and digital-twin studies in which updated physical models contribute to health assessment, fault-severity estimation, degradation tracking, remaining useful life prediction, or virtual–physical synchronization [29,30,31,32,33]. Only studies with an explicit relationship to physical modeling or model updating are treated as part of the principal FEMU framework.

This classification separates established FEMU applications from adjacent research that provides nonlinear models, uncertainty methods, computational tools, or operational frameworks. It also provides the organizational basis for the subsequent sections without requiring the same technical content to be repeated in this section.

### 2.4. Comparative Review Framework

The selected studies were compared using the following criteria:

1. Target system: rotor, bearing, shaft, blade, gearbox, or complete rotating machine;
2. Numerical model: beam, solid, reduced-order, nonlinear, stochastic, or hybrid model;
3. Measured response: natural frequency, mode shape, frequency response function, displacement, acceleration, shaft orbit, phase, or operational vibration;
4. Updated quantity: structural property, bearing coefficient, support parameter, damping property, clearance, friction, unbalance, or fault-related parameter;
5. Updating method: direct, sensitivity-based, optimization-based, surrogate-assisted, probabilistic, AI-driven, or hybrid;
6. Uncertainty treatment: deterministic, probabilistic, interval-based, or not explicitly considered;
7. Validation level: numerical verification, laboratory experiment, independent operating condition, or field measurement;
8. Computational requirement: offline, near-real-time, or real-time implementation;
9. Principal limitation: parameter correlation, non-uniqueness, model-form error, computational cost, measurement noise, data dependency, or limited generalization.

The evaluation therefore considers more than the minimum residual or accuracy reported by each study. Study quality was assessed qualitatively according to the clarity of the physical model and updating parameters, consideration of parameter identifiability and uncertainty, separation between calibration and validation, use of independent experimental or operational evidence, and reproducibility of the reported procedure. Application maturity was assessed according to the level of supporting evidence, ranging from numerical demonstration and laboratory experimental validation to independent or multi-condition validation and field or lifecycle implementation. These criteria were used consistently to distinguish methodological feasibility from experimentally demonstrated FEMU capability, rather than to assign a formal numerical quality score. Physical interpretability, parameter identifiability, model-form adequacy, uncertainty treatment, independent validation, and computational feasibility are also examined. These criteria are subsequently applied to the comparison of FEMU methods and rotating-system applications.

## 3. Sources of Model–Measurement Discrepancy in Rotating Machinery

Accurate finite element modeling of rotating machinery requires the numerical model to reproduce both the structural characteristics of the machine and the dynamic effects generated during operation. Nevertheless, discrepancies inevitably arise because numerical models rely on simplified geometry, nominal material properties, idealized interfaces, estimated support conditions, and incomplete representations of operating and fault mechanisms. These discrepancies are particularly important in rotating systems because their dynamic characteristics vary with rotational speed, bearing condition, thermal state, load, assembly condition, and fault severity [4,5,6,13,14,15,16,17,18].

The reliability of finite element model updating depends on identifying the dominant sources of discrepancy before selecting parameters for estimation. Increasing the number of adjustable parameters may reduce the calibration residual but can also produce correlated, non-unique, or physically unrealistic solutions. In contrast, parameters selected according to the physical mechanisms responsible for the observed discrepancy are more likely to retain interpretability and predictive capability [7,10,11,12].

**Figure 2:**
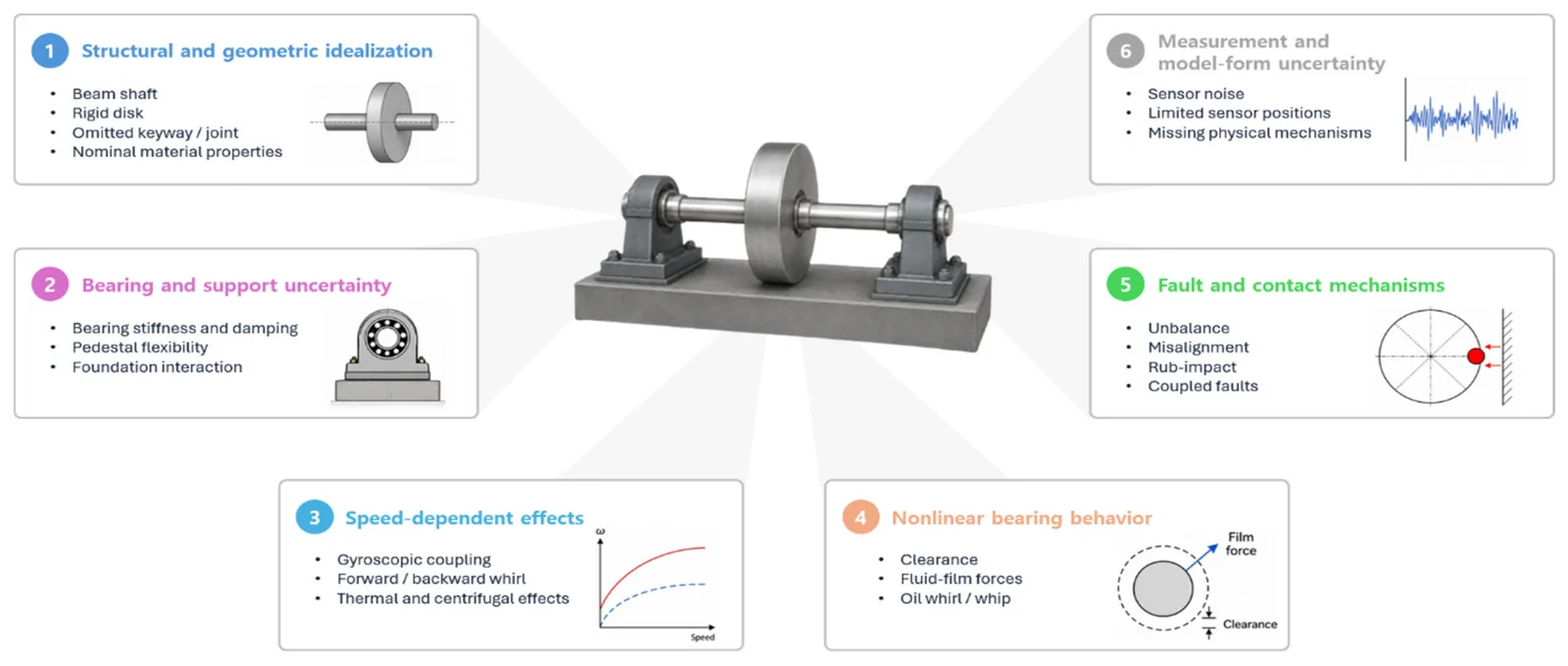
Major sources of model–measurement discrepancy and their effects on rotating-system responses.

Figure 2 summarizes the principal sources of model–measurement discrepancy considered in this review. These sources are grouped into structural and geometric idealization, bearing and support uncertainty, rotational and operating-condition dependence, nonlinear bearing behavior, fault-related interactions, parameter and model-form uncertainty, and measurement limitations. Their effects may appear in natural frequencies, critical speeds, frequency response functions, synchronous responses, shaft orbits, phase behavior, sub-synchronous vibration, and stability boundaries.

### 3.1. Structural and Geometric Idealization

Finite element models of rotating machinery commonly represent shafts using beam elements and disks using concentrated masses, rigid disks, or simplified solid elements. These representations reduce the number of degrees of freedom and make repeated dynamic analyses computationally feasible. However, simplified models may not reproduce local flexibility, distributed inertia, or the dynamic effects of geometric discontinuities [4,5,6].

Relevant sources of geometric discrepancy include stepped and tapered shaft sections, keyways, holes, grooves, shrink-fit connections, bolted joints, couplings, impellers, nonuniform mass distributions, and manufacturing tolerances. The importance of these details depends on the response quantity and frequency range considered. A beam-based shaft model may reproduce the first bending modes adequately while exhibiting larger discrepancies in higher modes or local deformation patterns. Likewise, a rigid-disk representation may capture the global rotor motion while neglecting disk flexibility and local coupling effects.

Material properties introduce additional uncertainty. Elastic modulus and density are often assigned using nominal values, whereas damping is commonly estimated because it arises from multiple mechanisms, including material dissipation, joint friction, bearing losses, foundation interaction, and surrounding-fluid effects. Temperature, residual stress, manufacturing variability, and aging may further modify the effective properties of the physical machine.

The required level of model fidelity should therefore be determined by the responses used for model correlation and by the intended prediction range. A numerical model does not need to reproduce every geometric detail, but it must contain sufficient physical fidelity to represent the dominant deformation, inertia, and energy-dissipation mechanisms relevant to the updating problem [4,5,6,7].

### 3.2. Bearing, Support, and Boundary-Condition Uncertainty

Bearing and support properties are often among the most uncertain quantities in a rotating-system model. The rotor is dynamically connected to rolling-element bearings or fluid-film bearings, housings, pedestals, foundations, couplings, and adjacent equipment, all of which can strongly influence natural frequencies, critical speeds, forced-response amplitudes, mode shapes, and stability behavior [13,14,34,35,36,37].

Supports are frequently represented using constant translational stiffness and damping coefficients. In physical machines, however, effective support characteristics may vary with rotational speed, static and dynamic load, preload, bearing clearance, lubrication condition, temperature, housing deformation, and foundation flexibility. Assembly tolerances and misalignment may further alter the effective boundary conditions.

Rolling-element bearing stiffness is governed by contact geometry, internal clearance, preload, load distribution, and contact angle. Consequently, nominal bearing properties may differ considerably from the effective coefficients of an assembled rotor system. Experimental rotor-model updating studies have therefore treated bearing stiffness and damping as identifiable physical parameters rather than fixed design values [13,34,35].

Journal bearings present an additional challenge because their dynamic behavior is generally represented using direct and cross-coupled stiffness and damping coefficients. These coefficients depend on rotational speed, static load, lubricant properties, bearing clearance, and the journal equilibrium position. Several FEMU studies have therefore adopted sequential or probabilistic procedures to separate structural uncertainty from bearing-coefficient uncertainty [14,36,37].

A central difficulty is that different physical parameters may generate similar response changes. Reduced shaft stiffness, reduced bearing stiffness, and increased pedestal flexibility may, for example, all shift natural frequencies or critical speeds in the same direction. Consequently, a measured discrepancy may indicate a support-related modeling error without uniquely identifying its physical source. This parameter correlation is one of the principal causes of ill-conditioning and non-unique estimates in rotor–bearing FEMU [7,12,37].

### 3.3. Rotational and Operating-Condition Dependence

Unlike stationary structures, rotating machinery contains dynamic effects that depend explicitly on rotational speed. Gyroscopic moments couple lateral motions in orthogonal directions and may separate rotor modes into forward- and backward-whirl branches as the rotational speed increases. Inadequate treatment of these effects can lead to incorrect predictions of critical speeds, whirl directions, and mode evolution [18,38].

Additional speed-dependent behavior may arise from centrifugal effects, bearing and seal coefficients, internal damping, fluid-induced forces, thermal expansion, preload variation, and changes in coupling conditions. Experimental model-updating studies using operational unbalance responses have demonstrated that parameter sensitivities and model discrepancies may vary significantly across rotational speeds [34,35,38]. Kang et al. estimated bearing dynamic coefficients and residual unbalance simultaneously in a double-disk rotor–bearing system using a dual augmented Kalman filter and steady-state unbalance responses [39]. The method was evaluated under different rotational speeds, measurement-noise levels, and measurement configurations, illustrating the value of operational response information for identifying parameters that interact under running conditions [39].

Load and temperature provide further sources of operating-condition dependence. Changes in transmitted torque, radial or axial load, lubricant temperature, and thermal expansion may modify shaft alignment, bearing clearance, contact conditions, and effective support stiffness. Consequently, a discrepancy observed at one operating point may arise from a change in operating state rather than from a permanent structural modification.

For this reason, rotating-machinery FEMU should not treat model discrepancy as independent of operating condition. Stationary natural frequencies, Campbell-diagram branches, synchronous vibration amplitudes, phase responses, shaft orbits, and stability thresholds provide different information about the underlying physical parameters. Multi-speed and multi-response measurements can therefore provide substantially greater parameter observability than calibration at a single operating point [14,34,35,37,38,39].

### 3.4. Nonlinear Bearing and Fluid-Film Effects

Linear rotor models are widely used because they permit efficient calculation of eigenvalues, critical speeds, and synchronous responses. However, linear assumptions become insufficient when bearing forces vary strongly with displacement, velocity, load, or operating state.

Hydrodynamic journal bearings are a representative source of nonlinear behavior. Linearized stiffness and damping coefficients describe small perturbations around an equilibrium position, but they may not reproduce large-amplitude motion, changes in journal position, or fluid-induced instability. Nonlinear fluid-film models are required to describe phenomena such as oil whirl, oil whip, amplitude-dependent responses, bifurcation, and changes in orbit geometry [17,18,40,41].

A systematic discrepancy between a linear model and measured responses may therefore originate from an inappropriate model form rather than from incorrect coefficient values. Adjusting constant bearing coefficients may improve agreement within a limited speed or amplitude range while failing to reproduce the response evolution outside that range.

Potential sources of nonlinear bearing discrepancy include radial clearance, lubricant properties, temperature-dependent viscosity, eccentricity ratio, cavitation assumptions, journal equilibrium position, and the formulation used to calculate fluid-film forces [17,40,41]. These quantities influence not only resonance amplitudes but also orbit shape, sub-synchronous components, instability onset, and stability boundaries.

Recent nonlinear rotor-bearing updating approaches have begun to incorporate nonlinear response formulations and analytical sensitivities into the parameter-estimation process [42]. Nevertheless, nonlinear FEMU remains substantially more difficult than conventional linear updating because the measured response may depend on amplitude, rotational speed, initial state, and response branch. Consequently, nonlinear model updating requires not only parameter estimation but also confirmation that the numerical model represents the correct physical response regime.

### 3.5. Rotor–Stator Contact, Misalignment, and Coupled Faults

Rotor–stator contact introduces nonlinear and potentially discontinuous forces when rotor displacement exceeds the available clearance. The resulting response depends on clearance, contact stiffness, friction, rotor eccentricity, stator support characteristics, rotational speed, and the relative motion between the rotating and stationary components [43,44,45,46].

Contact may be intermittent or continuous and can generate harmonics, subharmonics, impact-like waveforms, changes in orbit geometry, jump phenomena, and multiple stable response states. Numerical studies have further shown that uncertain contact parameters and initial conditions may produce different valid steady-state responses for the same nominal rotor configuration [43,44]. This behavior introduces a direct challenge for inverse identification because a parameter set producing a low residual may correspond to a response branch different from that observed experimentally.

Misalignment produces additional coupling through shaft bending, coupling forces, bearing-load redistribution, and changes in the shaft equilibrium position. It may also reduce the effective rotor–stator clearance and thereby increase the likelihood of rubbing. Experimental and numerical investigations of coupled misalignment–rubbing faults have demonstrated that the combined response cannot necessarily be interpreted as a simple superposition of the responses produced by the individual faults [45].

The same spectral component may therefore originate from different physical mechanisms. Elevated synchronous or second-harmonic vibration, for example, may be associated with unbalance, misalignment, support nonlinearity, coupling deformation, or intermittent rotor–stator contact. Agreement between the model and a single spectral component is consequently insufficient to demonstrate that the correct fault mechanism has been represented.

Compound faults further increase this ambiguity because one fault may alter the boundary or operating conditions under which another fault develops. Physically meaningful FEMU for such systems should therefore incorporate interacting fault mechanisms and use multiple response quantities, operating conditions, or transient responses to distinguish competing parameter combinations [43,44,45,46].

### 3.6. Parameter Uncertainty and Model-Form Error

Uncertainty in rotating-system models can be divided into parameter uncertainty, measurement uncertainty, and model-form uncertainty [10,11,12,15].

Parameter uncertainty refers to incomplete knowledge of quantities already represented in the model, including stiffness, damping, material properties, bearing clearance, lubricant properties, unbalance, or contact coefficients. Measurement uncertainty originates from sensor noise, mounting variability, finite frequency resolution, signal-processing procedures, and variations in operating conditions during testing.

Model-form uncertainty occurs when the assumed physical representation is incomplete or inappropriate. Examples include representing a nonlinear bearing using constant coefficients, neglecting pedestal flexibility, excluding thermal effects, simplifying coupling behavior, or omitting rotor–stator contact.

This distinction is particularly important in FEMU because parameter adjustment cannot fully compensate for a missing physical mechanism. An apparent error in bearing stiffness may actually originate from foundation flexibility, temperature-dependent lubricant behavior, nonlinear fluid forces, or coupling deformation. Optimization may nevertheless assign an apparently plausible parameter value that reproduces the calibration data while reducing predictive reliability under other operating conditions.

Rotor-support systems provide a representative example because effective stiffness and damping may remain uncertain even when the physical form of the support is known. Probabilistic model updating has been used to characterize uncertainty in identifiable rotor-support parameters [37], while interval model-validation approaches have been developed to estimate credible ranges of uncertain support stiffness and damping [47]. He et al. proposed an interval model-validation method for rotor-support systems using a k-means Bayesian procedure [47]. The uncertain support stiffness and damping were represented using interval parameters, allowing model validation to address credible parameter ranges rather than only a single deterministic optimum [47].

Probabilistic rotor-model updating can quantify uncertainty in identifiable parameters [37], while nonlinear formulations can extend the parameterization to more realistic force models [42]. However, uncertainty associated with an inadequate model form should not be interpreted solely as parameter uncertainty. In practice, potential model-form inadequacy can be investigated by examining persistent residual patterns across different rotational speeds, loads, or independent response quantities, comparing alternative physical model formulations, and assessing whether the identified parameters remain physically plausible and consistent across operating conditions. Systematic residuals that persist after parameter updating, or parameter estimates that vary unrealistically between operating conditions, may indicate that an important physical mechanism is missing or inadequately represented. Parameter estimation and model-form assessment should therefore remain distinct components of the FEMU process [10,11,12,15].

### 3.7. Measurement Limitations and Incomplete Observability

FEMU relies on experimentally measured quantities such as natural frequencies, damping ratios, mode shapes, frequency response functions, shaft displacement, acceleration spectra, shaft orbits, phase, and operational vibration responses. The information content and uncertainty of these measurements determine which physical parameters can be identified reliably.

Industrial measurements are commonly affected by limited sensor locations, electrical and mechanical noise, mounting variability, inconsistent excitation, insufficient frequency resolution, missing or closely spaced modes, and differences between numerical and experimental degrees of freedom. Operational measurements may additionally vary with rotational speed, load, temperature, and environmental conditions [48].

Limited sensor placement reduces observability because different parameter combinations may produce similar responses at the available measurement locations. Increasing the number of adjustable parameters without increasing independent measurement information therefore worsens the inverse problem. The number of parameters that can be estimated reliably is constrained not simply by the number of measurements, but by the independence and parameter sensitivity of those measurements [7,12].

Different response quantities also exhibit different uncertainty characteristics. Natural frequencies are generally more repeatable than damping estimates, whereas mode-shape components depend strongly on sensor location and mode pairing. Frequency response functions provide both amplitude and phase information but are sensitive to excitation quality and boundary-condition repeatability. Operational responses contain direct information from the rotating machine but may combine the effects of structural, support, excitation, operating, and fault parameters.

Sensor placement, excitation conditions, and rotational-speed selection influence parameter identifiability through different mechanisms. Sensor placement primarily determines spatial observability; measurements obtained near parameter-sensitive locations can improve discrimination, whereas measurements near response nodes or locations with similar parameter sensitivities may increase correlation among candidate parameters. Excitation conditions determine which dynamic mechanisms are activated: controlled modal excitation is particularly useful for structural and support-related properties, whereas operational unbalance excitation provides information on speed-dependent bearing, support, and excitation parameters. For nonlinear parameters such as clearance or contact stiffness, the excitation level must also be sufficient to activate the corresponding nonlinear response. Rotational-speed selection provides an additional means of separating parameters because response sensitivities vary with speed; therefore, multi-speed measurements, particularly around resonance, critical-speed, or stability-sensitive regions, can reduce ambiguities that remain under a single operating condition [7,12,14,34,35,37,38,39,49,50].

Measurement uncertainty should therefore be considered explicitly when interpreting model discrepancy. A residual may represent a physical model error, an operating-condition change, or insufficient measurement quality. These possibilities should be evaluated before uncertain model parameters are adjusted.

### 3.8. Implications for FEMU Parameter Selection

The discrepancy sources described above demonstrate that parameter selection is fundamentally a physical-modeling and identifiability problem rather than only a numerical optimization problem. Updating all uncertain quantities is neither computationally efficient nor physically reliable. Candidate parameters should instead be selected according to the mechanisms most likely to produce the observed model–measurement discrepancy [7,12,51].

A suitable parameter-selection procedure should:

1. identify the response characteristics that differ between the numerical model and the physical system;
2. relate each discrepancy to plausible structural, support, operating, or fault mechanisms;
3. select candidate parameters associated with those mechanisms;
4. evaluate the sensitivity and independence of the selected responses with respect to those parameters across candidate sensor locations, excitation conditions, and rotational speeds;
5. remove parameters with negligible sensitivity or strong correlation;
6. impose bounds consistent with geometry, material properties, bearing design, assembly conditions, and operating states; and
7. determine whether the remaining discrepancy is attributable to parameter uncertainty or inadequate model form.

The response quantities used for updating should correspond to the physics of the candidate parameters. Structural modal discrepancies may primarily indicate uncertainty in shaft, joint, or support stiffness. Errors in critical speed or synchronous response may indicate uncertainty in bearing properties, support characteristics, damping, or unbalance. Sub-synchronous components, stability boundaries, and orbit changes may require nonlinear fluid-film representations, whereas impact-like responses, higher harmonics, jump behavior, and hysteresis may indicate contact or interacting fault mechanisms.

Accordingly, physically meaningful rotating-machinery FEMU should begin with discrepancy interpretation and parameter screening before numerical estimation. The objective is not to identify the parameter combination that produces the smallest calibration residual, but to obtain an identifiable and physically plausible model that remains predictive across independent responses and operating conditions.

## 4. Classification and Comparative Analysis of FEMU Methods

Finite element model updating can be formulated as an inverse problem in which uncertain physical parameters are estimated from discrepancies between numerical predictions and measured responses. Although FEMU methods differ in their mathematical formulations and numerical procedures, their common objective is not merely to minimize model–measurement discrepancies but to obtain a numerical model that retains physical interpretability and predictive capability beyond the calibration data.

For a rotating system, the governing equation may be expressed as

(1) $$M\left(p\right)\;\ddot{x}+\left[C\left(p\right)+\Omega G\left(p\right)\right]\dot{x}+K\left(p,\Omega \right)x=f\left(t\right)$$

where M, C, G, and K are the mass, damping, gyroscopic, and stiffness matrices, respectively; Ω is the rotational speed; x is the response vector; and p is the vector of uncertain model parameters.

The general FEMU problem can be expressed as

(2) $$\hat{p}=\underset{p}{arg\;min}\;J\left(p\right)$$

where $J\left(p\right)$ is an objective function representing the discrepancy between measured and calculated responses. A commonly used weighted least-squares form is

(3) $$J\left(p\right)={\left[y_{m}-y_{FE}\left(p\right)\right]}^{T}W\left[y_{m}-y_{FE}\left(p\right)\right]$$

where $y_{m}$ and $y_{FE}$ denote the measured and finite-element responses, respectively, and W is a weighting matrix representing the relative importance, scale, or confidence of the selected response quantities.

For rotating machinery, this inverse problem is complicated by speed-dependent dynamics, correlated bearing and support parameters, limited measurement information, nonlinear response mechanisms, and changes in operating conditions. Consequently, the updating algorithm should not be considered independently from candidate-parameter selection, response selection, parameter identifiability, uncertainty treatment, and validation.

**Figure 3:**
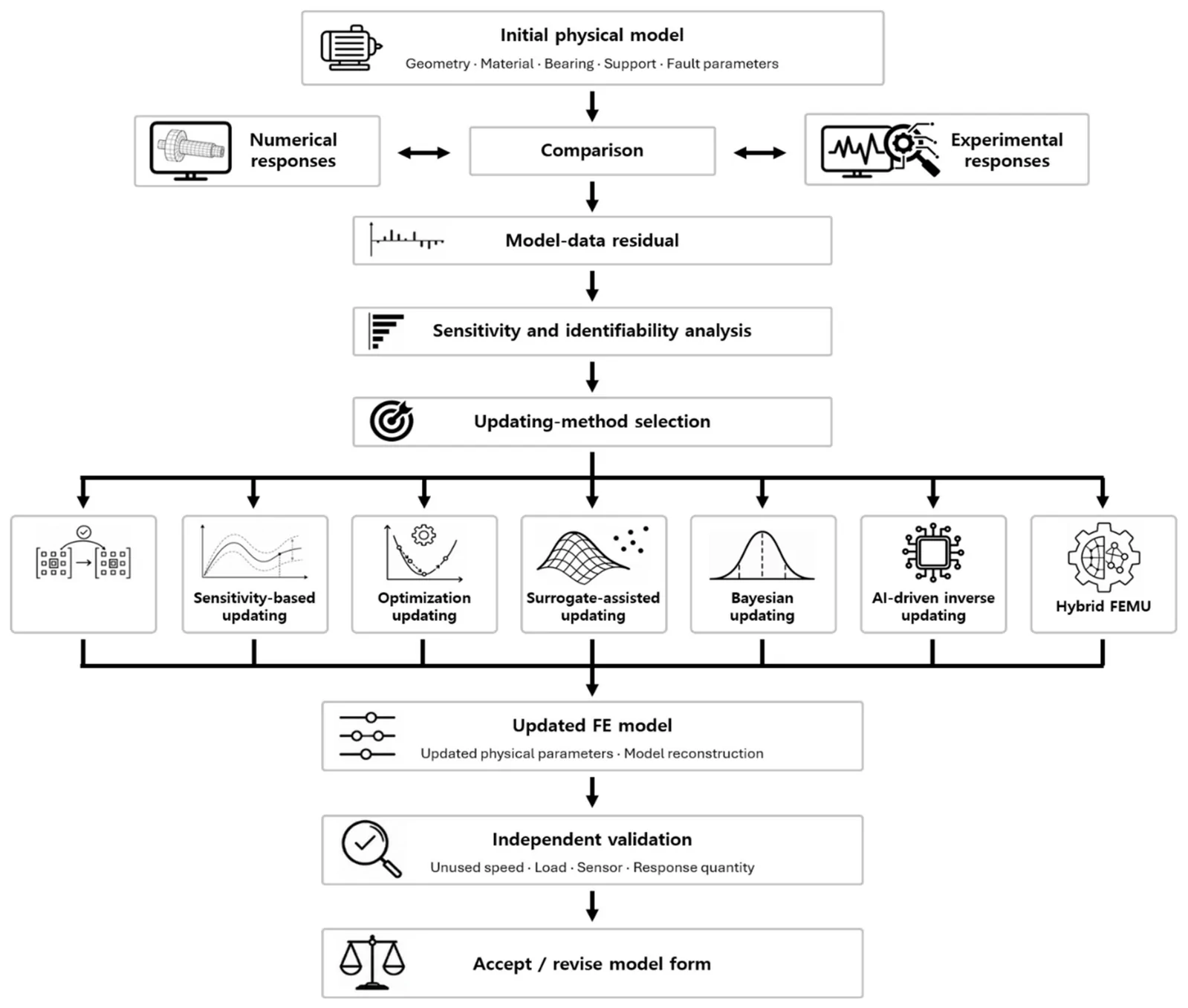
General workflow and methodological classification of rotating-machinery FEMU.

In this review, FEMU methods are classified as direct matrix correction, sensitivity-based updating, optimization-based updating, surrogate-assisted updating, Bayesian and probabilistic updating, AI-driven inverse updating, and hybrid strategies [4,5,6,7,8,9,10,11,12,19]. Figure 3 places these methods within a common workflow comprising model–measurement comparison, candidate-parameter selection, sensitivity and identifiability assessment, parameter estimation, reconstruction of the physical model, and independent validation.

The classification concerns how uncertain parameters are estimated. The physical causes of discrepancy were discussed in Section 3, while representative rotating-system applications and validation results are reviewed separately in Section 5.

### 4.1. Direct Matrix Correction Methods

Direct matrix correction methods modify the assembled mass, stiffness, or damping matrices so that selected numerical modal properties reproduce their experimental counterparts. Corrections are generally obtained from measured eigenvalues, mode shapes, orthogonality conditions, or equations of motion without repeatedly evaluating a physically parameterized finite element model [4,5,6].

The principal advantage of direct correction is computational efficiency. When sufficiently complete and accurately paired modal data are available, substantial reductions in modal discrepancy can be achieved with relatively few numerical operations. Direct methods can therefore be useful for preliminary numerical correlation and for identifying portions of the assembled matrices associated with large model–measurement discrepancies.

However, the corrected matrix terms do not necessarily correspond to physically measurable quantities such as shaft stiffness, bearing stiffness, support flexibility, material properties, or damping. Matrix-level modifications may also alter sparsity, connectivity, symmetry, or positive definiteness. Consequently, a directly corrected model may reproduce the modal quantities used during updating while providing limited physical interpretation and uncertain predictive capability outside the calibration conditions [4,5,6].

This limitation is particularly important for rotating machinery because model updating is frequently intended to estimate bearing, support, coupling, structural, or fault-related parameters that are subsequently used to predict critical speeds, forced responses, whirl behavior, or stability. Direct matrix correction is therefore more suitable for mathematical model correlation and preliminary discrepancy localization than for physically interpretable parameter identification.

### 4.2. Sensitivity-Based Updating Methods

Sensitivity-based FEMU approximates the change in calculated responses caused by small variations in selected model parameters. Around the current parameter state $p_{k}$, the finite element response can be locally linearized as

(4) $$y_{FE}\left(p_{k}+\Delta p\right)\approx y_{FE}\left(p_{k}\right)+S_{k}\Delta p$$

where $S_{k}$ is the sensitivity or Jacobian matrix defined as

(5) $$S_{k}={\left.\frac{\partial y_{FE}}{\partial p}\right|}_{p=p_{k}}$$

The parameter correction may then be obtained using a regularized least-squares formulation:

(6) $$\Delta \hat{p}=\underset{\Delta p}{arg\;min}{∥W_{r}^{1/2}\left[r_{k}-S_{k}\Delta p\right]∥}_{2}^{2}+\lambda {∥W_{p}^{1/2}\Delta p∥}_{2}^{2}$$

where $r_{k}$ is the current model–measurement residual, $W_{r}$ represents the weighting of the measured responses, $W_{p}$ penalizes excessive parameter changes, and λ is the regularization coefficient.

Sensitivity-based FEMU is computationally efficient when the number of candidate parameters is moderate and the initial numerical model is reasonably close to the physical system. It also maintains an explicit relationship between changes in measured responses and variations in physical parameters. Analytical, semi-analytical, or finite-difference sensitivities may be used depending on model complexity and software accessibility [4,7].

The principal limitation is the local nature of the linear approximation. When the initial discrepancy is large or the parameter–response relationship is strongly nonlinear, the sensitivity matrix evaluated at the current parameter state may not represent the actual response variation adequately. Iterative recalculation of the sensitivity matrix is then required.

Ill-conditioning is another major concern. If two parameters produce similar changes in the measured responses, their sensitivity vectors become highly correlated, and the corresponding parameters cannot be estimated independently with sufficient reliability. Regularization, parameter grouping, singular-value decomposition, engineering constraints, and elimination of low-sensitivity parameters can be used to improve numerical stability [7,10,11,12].

This issue is particularly relevant to rotor–bearing systems because shaft stiffness, bearing stiffness, support flexibility, and damping can produce similar changes in modal and operational responses. Candidate parameters should therefore be screened according to their sensitivity and physical relevance before updating [13,14].

The sensitivity matrix should also reflect the operating conditions relevant to the estimated parameters. Sensitivities calculated from stationary modal data may provide insufficient information for parameters whose effects vary with rotational speed. Multi-speed responses can improve parameter observability because the influence of bearing, support, and gyroscopic parameters may change near resonances and critical speeds.

### 4.3. Optimization-Based Updating Methods

Optimization-based FEMU treats parameter estimation as a constrained search problem. The finite element model is evaluated repeatedly for candidate parameter sets, and an objective function is used to quantify the discrepancy between numerical and experimental responses [8,34,38,52,53].

For rotating machinery, different response quantities may be combined within a multi-response objective function:

(7) $$J\left(p\right)=w_{f}J_{f}\left(p\right)+w_{\phi }J_{\phi }\left(p\right)+w_{FRF}J_{FRF}\left(p\right)+w_{o}J_{o}\left(p\right)$$

where $J_{f}$, $J_{\phi }$, $J_{FRF}$, and $J_{o}$ represent residual terms associated with natural frequencies, mode shapes, frequency response functions, and operational responses, respectively. The corresponding weighting factors should account for differences in response magnitude, measurement reliability, and relevance to the physical parameters being estimated.

When mode shapes are included, their correlation can be evaluated using the modal assurance criterion (MAC):

(8) $$MAC\left(\phi_{m},\phi_{FE}\right)=\frac{{\left|\phi_{m}^{H}\phi_{FE}\right|}^{2}}{\left(\phi_{m}^{H}\phi_{m}\right)\left(\phi_{FE}^{H}\phi_{FE}\right)}$$

where $\phi_{m}$ and $\phi_{FE}$ denote the measured and calculated mode-shape vectors, respectively, and the superscript H denotes the conjugate transpose.

Optimization algorithms may be classified broadly as gradient-based or derivative-free methods. Gradient-based approaches generally converge efficiently when the objective function is smooth and a suitable initial parameter estimate is available. However, they can be sensitive to local minima, poor conditioning, and inaccurate initial values.

Derivative-free, evolutionary, and population-based approaches can explore broader regions of the parameter space and accommodate nonlinear, nonsmooth, or multimodal objective functions. Their principal disadvantage is the substantially larger number of numerical model evaluations required [8,34,35,52].

Optimization-based strategies have been applied to rotor and rotor–bearing systems for estimating shaft, bearing, support, damping, and unbalance-related parameters using modal and operational responses [14,34,35,38,39,52]. Optimization-based FEMU is particularly useful when several response quantities must be considered simultaneously, analytical sensitivities are unavailable, or the parameter–response relationship is strongly nonlinear. Physical bounds based on geometry, material properties, bearing design, clearances, and assembly tolerances can also be incorporated explicitly into the optimization problem.

The principal limitations are computational cost and solution non-uniqueness. Different combinations of structural, bearing, support, and damping parameters may generate similar values of the objective function. Therefore, obtaining the lowest calibration residual does not necessarily demonstrate that the physically correct parameter set has been identified.

Optimization-based updating should consequently be preceded by parameter-sensitivity and identifiability analysis and followed by physical plausibility assessment and independent validation. For rotating machinery, the preferred solution is not necessarily the parameter combination producing the smallest residual but the one that remains physically reasonable and predictive under responses or operating conditions that were not used during calibration.

### 4.4. Surrogate-Assisted Updating

Surrogate-assisted FEMU reduces the computational cost associated with repeated evaluation of high-fidelity finite element models. Instead of evaluating the original numerical model during every optimization or uncertainty-analysis step, an approximate relationship is constructed between uncertain parameters and selected responses.

Representative approaches include response-surface models, radial-basis functions, Kriging, Gaussian-process regression, and reduced-order models [9,35,49,50]. When only a limited number of high-fidelity numerical samples are available, Kriging and Gaussian-process models are particularly useful because they can provide both response approximation and information on prediction uncertainty, which can support adaptive sampling [35,49,50]. In contrast, more highly parameterized data-driven surrogate models generally require broader training coverage but can provide very rapid repeated evaluation after training. Therefore, surrogate selection should consider not only approximation accuracy but also sample availability, response nonlinearity, uncertainty representation, and the required number of repeated evaluations.

Kriging-based surrogate models have been applied to bearing-parameter identification in rotor–bearing systems [35], while adaptive Gaussian-process models have been used for operational-response-based rotor model updating [49]. More recently, adaptive hybrid-kernel Gaussian-process models have been incorporated into Bayesian rotor-model updating [50].

A typical surrogate-assisted FEMU procedure consists of:

1. defining the admissible physical-parameter domain;
2. generating parameter samples within the defined domain;
3. evaluating the original finite element model at the sampled points;
4. constructing and verifying the surrogate model;
5. performing optimization or uncertainty analysis using the surrogate;
6. reconstructing and validating the final parameter estimate using the original finite element model.

The principal advantage is computational efficiency. Once constructed, the surrogate can be evaluated rapidly during global optimization, Monte Carlo sampling, Bayesian inference, or repeated parameter estimation. This advantage becomes important when the original model requires multi-speed analyses, large finite element systems, or nonlinear time-domain simulations.

The principal limitation is dependence on the parameter and operating domains represented by the training samples. Rotor responses can vary rapidly near critical speeds, instability boundaries, bifurcation points, and contact transitions. A surrogate that provides low average prediction error across the overall parameter domain may still exhibit substantial local error in regions that are important for parameter identification.

Adaptive sampling can improve reliability by adding numerical samples in regions characterized by high approximation uncertainty or large response gradients. Local surrogate models may also be more appropriate than a single global approximation when strongly nonlinear response regimes are involved.

Two different levels of validation should be clearly distinguished. Surrogate verification determines whether the approximate model reproduces unused results from the original numerical model. Physical-model validation determines whether the final updated finite element model reproduces independent experimental measurements. Accurate approximation of the numerical solver alone does not demonstrate that the physical model is correct.

### 4.5. Bayesian and Probabilistic Updating Methods

Deterministic FEMU generally provides a single estimated parameter vector, whereas Bayesian and probabilistic approaches represent uncertain parameters using probability distributions.

A Bayesian updating problem may be expressed as

(9) $$\pi \left(p|D\right)=\frac{L\left(D|p\right)\;\pi_{0}\left(p\right)}{Z}$$

where $\pi_{0}\left(p\right)$ is the prior distribution of the uncertain parameter vector p, $L\left(D|p\right)$ is the likelihood of the measured data D for a specified parameter vector, $\pi \left(p|D\right)$ is the posterior distribution, and Z is the normalization constant or model evidence [7,10,11,12].

The prior distribution represents available engineering knowledge, manufacturing tolerances, or physically admissible parameter ranges. The likelihood quantifies agreement between the measured and calculated responses while accounting for measurement uncertainty. The posterior distribution therefore provides information regarding both the estimated parameters and their remaining uncertainty.

This capability is particularly relevant to rotating machinery because several combinations of bearing, support, structural, and damping parameters may produce similar measured responses. Bayesian updating can represent this ambiguity explicitly instead of forcing the inverse problem to provide only one deterministic parameter estimate.

Bayesian FEMU is therefore useful when measurements are noisy, parameter identifiability is limited, or uncertainty in the updated model is itself an important result. Posterior parameter uncertainty may subsequently be propagated to quantities such as critical speeds, response amplitudes, and stability boundaries.

Bayesian and probabilistic approaches have been applied to rotor–bearing–support systems for estimating uncertain support parameters and quantifying posterior parameter dependence [37]. Interval-based validation has also been used to characterize credible ranges of uncertain rotor-support parameters [47], while Gaussian-process-assisted Bayesian updating has recently been applied to measured unbalance responses of rotor systems [50].

The principal limitation is computational demand. Posterior estimation using Markov chain Monte Carlo or related sampling methods can require a large number of numerical model evaluations. High-fidelity rotor models may therefore require surrogate or reduced-order models for practical probabilistic updating.

Bayesian results are also influenced by the assumed prior distribution, likelihood function, and residual covariance. An excessively restrictive prior can dominate the information contained in the measurements, whereas an excessively broad prior may preserve weak identifiability and increase computational requirements.

Furthermore, model-form error should not be represented solely as parameter uncertainty. If an important physical mechanism is absent from the numerical model, probabilistic inference may produce a narrow posterior distribution around physically misleading parameter values. Bayesian updating should therefore be accompanied by model-form assessment and independent validation.

### 4.6. AI-Driven Inverse Updating

A direct example of AI-based physical-parameter updating is provided by Noever-Castelos et al. [19], who used an invertible neural-network formulation to estimate finite element parameters from structural response information. More generally, neural networks, encoder–decoder models, probabilistic or invertible architectures, and physics-guided approaches can serve as inverse estimators when their outputs are defined as identifiable physical model parameters rather than diagnostic class labels.

In contrast, References [20,21,22,23,24,25,26,27,28] do not directly demonstrate physical-parameter updating and are therefore treated as FEMU-enabling studies rather than direct FEMU evidence. Their relevance lies in techniques such as vibration-feature learning, robustness to noise and varying operating conditions, simulation-generated training data, synthetic-data augmentation, transfer learning, and domain adaptation. These capabilities may support future AI-assisted FEMU, but physical-parameter updating would additionally require the estimated quantities to correspond to identifiable model parameters, to be reintroduced into the finite element model, and to be validated against independent experimental responses.

AI-driven FEMU uses datasets containing physical parameters and corresponding numerical or measured responses to learn forward or inverse parameter–response relationships.

In a forward configuration, a trained AI model approximates the response of the finite element model and can subsequently be incorporated into optimization or probabilistic inference. In an inverse configuration, measured responses are provided directly to the trained model to estimate uncertain physical parameters.

Neural networks, encoder–decoder architectures, invertible neural networks, and physics-guided learning approaches may be formulated for inverse physical-parameter estimation, although direct FEMU evidence among the reviewed AI studies is represented primarily by Reference [19]. Their suitability depends on the characteristics of the inverse problem and the available training data. Conventional neural-network or encoder–decoder models are useful for rapid deterministic estimation when the parameter–response relationship is sufficiently represented by the training database, whereas probabilistic or invertible architectures are more appropriate when multiple parameter combinations can produce similar responses. Physics-guided approaches can further constrain the estimated relationship using known physical behavior and parameter bounds. Parametric finite element simulations generally provide the principal training database and may be supplemented with experimental measurements [19], while the simulation-to-real and data-augmentation strategies reported in References [20,21,22,23,24,25,26,27,28] provide complementary enabling techniques rather than direct physical-parameter updating evidence.

The main advantage is rapid inference after the offline simulation and training stages have been completed. AI-based inverse models can approximate complex nonlinear parameter–response relationships and may therefore support repeated parameter estimation when direct finite element optimization is computationally expensive.

However, the resulting inverse relationship is reliable primarily within the parameter and operating domains represented by the training database. Parameter estimates may become unreliable when measured responses correspond to operating conditions, machine configurations, fault mechanisms, or parameter combinations not represented during training.

Training data generated solely from numerical simulations also inherit the assumptions and model-form errors of the original numerical model. High prediction accuracy against simulated data therefore does not demonstrate that the inferred parameters accurately represent the physical machine. To reduce this simulation-to-experiment gap, the numerical training domain should include realistic variations in operating conditions, physical parameters, and measurement characteristics. When experimental data are available, fine-tuning, hybrid simulation–experimental training, or domain-adaptation strategies can also be used to improve transfer to the physical system, followed by validation using experimental responses excluded from training.

Other important limitations include simulation-to-experiment discrepancy, sensor variability, operating-condition variation, parameter correlation, and hidden non-identifiability. A deterministic neural network may return a unique parameter estimate even when several physical parameter combinations can reproduce the same measured response.

Probabilistic and invertible architectures can represent such non-unique inverse relationships more explicitly. Nevertheless, AI-generated parameter estimates should be treated as candidate physical parameters rather than automatically accepted model corrections.

The estimated parameters should satisfy physically meaningful bounds and should ultimately be reintroduced into the original finite element model. The reconstructed model should then be evaluated against experimental responses that were not used during training or parameter estimation. AI-assisted inverse analysis should therefore accelerate or support FEMU rather than replace physical-model validation.

### 4.7. Hybrid FEMU Strategies

No single FEMU method is optimal for all rotating-machinery applications. Hybrid strategies combine complementary approaches to balance physical interpretability, parameter identifiability, numerical robustness, uncertainty treatment, and computational efficiency.

Representative combinations include:

- sensitivity analysis followed by constrained optimization [14];
- global optimization accelerated by Kriging or Gaussian-process models [35,49];
- surrogate-assisted Bayesian inference [50];
- deterministic parameter estimation followed by uncertainty propagation [12,15];
- physics-based finite element models combined with AI-based inverse estimators [19];
- high-fidelity offline updating followed by reduced-order or surrogate-assisted repeated estimation [35,49,50].

A practical hybrid FEMU workflow begins with a physically parameterized rotor model and a limited set of candidate parameters. Sensitivity and parameter-correlation analyses are first used to identify weakly observable or redundant parameters. Optimization, Bayesian inference, or inverse learning is then performed within the reduced parameter space.

Surrogate or reduced-order models may subsequently be introduced when repeated evaluation of the high-fidelity finite element model becomes computationally prohibitive. Regardless of the computational method used for estimation, the resulting physical parameters should ultimately be reconstructed in the original finite element model and validated using independent responses or operating conditions.

The principal strength of hybrid FEMU lies in the separation of methodological roles. Physics-based modeling defines admissible mechanisms and parameters; sensitivity analysis evaluates identifiability; optimization or probabilistic inference estimates the parameters; and surrogate or AI methods reduce computational cost.

Combining these approaches does not eliminate the need for physically meaningful parameter bounds, model-form assessment, uncertainty evaluation, or independent experimental validation.

### 4.8. Comparative Assessment and Method Selection

Table 1 summarizes the principal characteristics of the FEMU approaches considered in this review.

Method selection should not be based solely on the smallest calibration residual. For rotating machinery, the following factors should be considered jointly:

- physical meaning of the updated parameters;
- parameter sensitivity and identifiability;
- type and quality of the available measurements;
- dependence of the response on rotational speed and operating conditions;
- linear or nonlinear characteristics of the parameter–response relationship;
- robustness to measurement and parameter uncertainty;
- computational resources and required update frequency;
- predictive accuracy under responses or operating conditions not used during calibration.

Sensitivity-based updating is suitable for relatively low-dimensional problems in which candidate parameters are identifiable and the initial numerical model is reasonably close to the measured system. Optimization-based methods are preferable when multiple response quantities must be combined or when the parameter–response relationship is nonlinear. Surrogate-assisted methods are advantageous when repeated finite element evaluations dominate the computational cost. Bayesian approaches are appropriate when uncertainty in the estimated parameters or model predictions is itself an important result. AI-driven inverse approaches become attractive when a sufficiently representative simulation and experimental database is available and rapid repeated estimation is required.

For nonlinear, high-dimensional, or operationally varying rotor systems, hybrid strategies provide a practical means of assigning different roles to physics-based modeling, sensitivity analysis, optimization, uncertainty quantification, and computational acceleration. Regardless of the method selected, however, the final criterion should remain predictive credibility. The estimated parameters should be identifiable and physically plausible, and the reconstructed numerical model should reproduce independent experimental responses beyond those used during calibration.

## 5. Applications of FEMU in Rotating Systems

Finite element model updating has been applied to rotating machinery primarily to estimate physical parameters that are difficult to determine directly and to improve the predictive accuracy of operational dynamic responses. Representative updating targets include shaft and support properties, bearing stiffness and damping, journal-bearing dynamic coefficients, material and structural parameters, unbalance-related quantities, and other parameters governing rotor-dynamic behavior.

The reviewed applications can be divided into two principal groups. The first group comprises direct FEMU studies, in which measured responses are used to estimate or correct physical parameters of a rotating-system numerical model. The second group comprises FEMU-enabling studies, which establish nonlinear physical models, uncertainty-analysis procedures, surrogate models, or coupled-fault representations that can subsequently be incorporated into an updating framework but do not themselves complete experimental physical-parameter updating.

This distinction is important because agreement between a forward numerical model and measured vibration characteristics does not itself constitute FEMU. A direct FEMU study should contain an inverse relationship in which experimental information modifies or estimates one or more parameters of the physical model.

**Figure 4:**
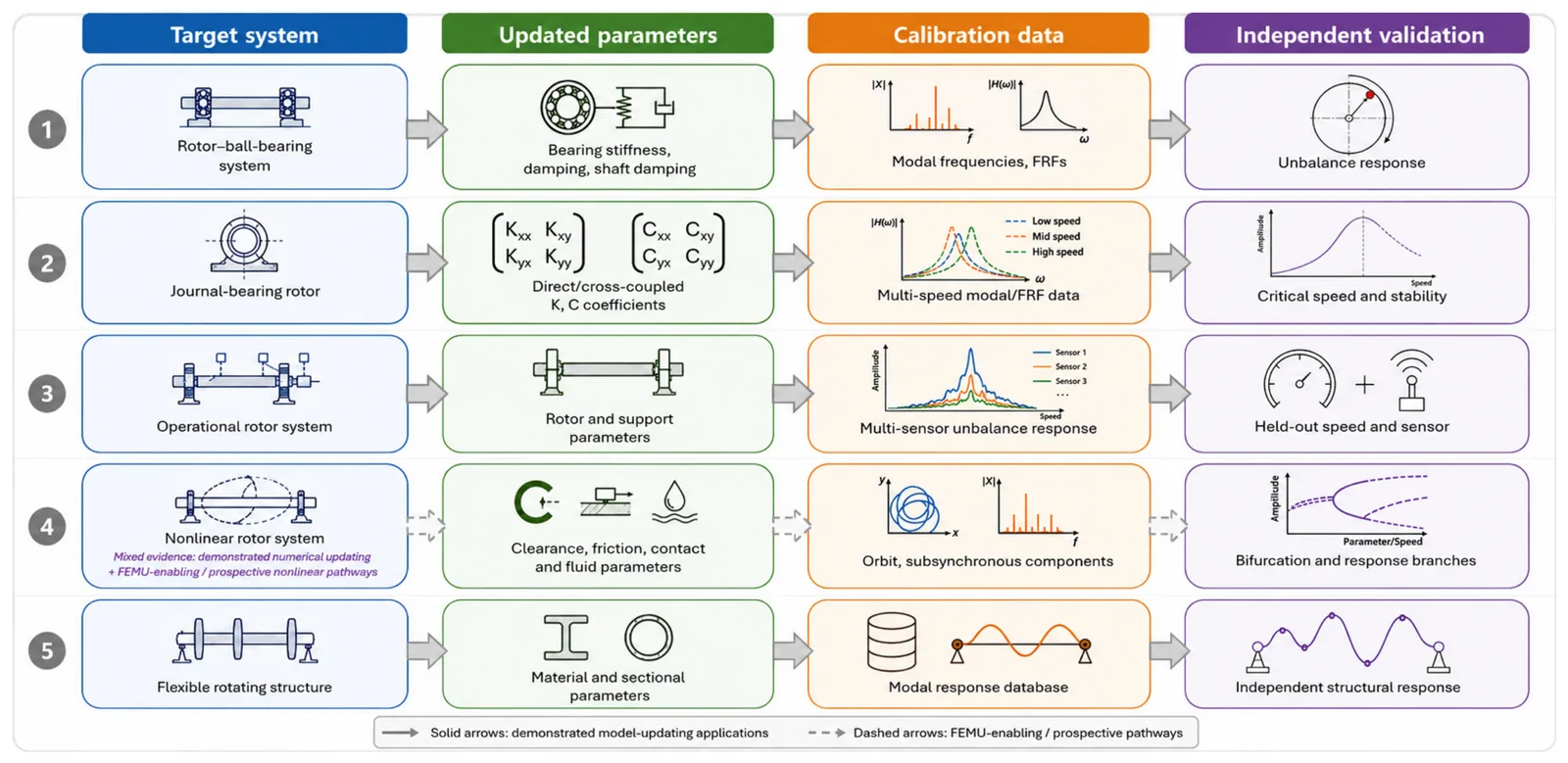
Literature-based synthesis of rotating-system FEMU applications, updated parameters, calibration measurements, and validation responses. Solid arrows indicate demonstrated model-updating applications, whereas dashed arrows indicate FEMU-enabling or prospective nonlinear updating pathways.

Figure 4 provides a literature-based synthesis of the rotating-system FEMU applications reviewed in this section. Demonstrated model-updating applications are represented by solid pathways, whereas the nonlinear rotor pathway is distinguished using dashed arrows to indicate that the reviewed evidence includes FEMU-enabling or prospective nonlinear updating approaches in addition to demonstrated numerical nonlinear updating.

### 5.1. Rotor–Bearing Parameter Identification and Model Calibration

Rotor–bearing systems represent one of the most established application areas of rotating-machinery FEMU because bearing and support parameters are difficult to determine accurately from nominal design information alone. Their effective values depend on installation, preload, internal clearance, support flexibility, assembly condition, and operating state.

Chouksey et al. investigated model updating of a ball-bearing-supported rotor system [13]. The uncertain parameters included bearing stiffness and damping together with shaft material damping. An inverse eigen-sensitivity approach was used to estimate the selected parameters from experimental modal information. Unlike direct matrix correction, the procedure retained the physical parameterization of the rotor model.

An important aspect of this study was that model evaluation was not restricted to the modal quantities used during calibration. The updated model was also assessed using frequency response functions and subsequently employed for unbalance-response prediction and rotor balancing. The application therefore illustrates an important principle of FEMU: the usefulness of an updated model should be judged by its ability to predict responses beyond those directly used for parameter estimation.

Kim et al. proposed a bearing-parameter identification method based on measured unbalance responses of a rotor–bearing system [34]. Bearing parameters and disk-unbalance information were treated as unknown variables, and a clustering-based hybrid evolutionary algorithm was employed to perform global parameter estimation.

This approach is significant because operational unbalance responses contain information directly related to the running rotor rather than only to its stationary modal characteristics. However, the use of operational responses also increases parameter correlation because bearing properties, unbalance magnitude and phase, shaft properties, and support characteristics can influence the measured response simultaneously.

Han et al. subsequently introduced a Kriging-surrogate-assisted approach for bearing-parameter identification [35]. Finite element simulations were used to construct a parameter–response database, while a Kriging surrogate approximated the original numerical relationship. Differential evolution was then used to estimate the unknown parameters from the measured responses.

This study illustrates the transition from conventional repeated finite element optimization toward surrogate-assisted FEMU. The underlying updating target remained physically interpretable bearing parameters, whereas the surrogate was used primarily to reduce the computational burden associated with repeated model evaluations.

Jha et al. applied nonlinear least-squares optimization to rotor-system model updating [38]. The formulation minimized discrepancies between measured and predicted rotor responses while adjusting selected physical parameters. This study demonstrates that conventional nonlinear optimization remains applicable when the candidate parameter set is sufficiently constrained and the relationship between physical parameters and measured responses can be represented using an appropriate objective function.

More recently, Kang et al. developed a simultaneous parameter-identification procedure for a double-disk rotor–bearing system using a dual augmented Kalman filter [39]. The method estimated bearing dynamic coefficients and residual unbalance from steady-state unbalance responses without requiring external excitation. Sensitivity analysis was used to examine the coupling between the unknown quantities, and the approach was evaluated numerically and experimentally under different rotational speeds, measurement-noise levels, and measurement configurations [39].

The Kang et al. study is particularly relevant to operational FEMU because it demonstrates that bearing and excitation-related parameters can be estimated simultaneously from running-condition measurements. At the same time, the reported coupling between residual unbalance and bearing coefficients reinforces the need for parameter-identifiability analysis before interpreting estimated values as unique physical quantities [39].

Collectively, these investigations demonstrate that rotor–bearing FEMU and related physical parameter identification can be performed using modal properties, frequency response functions, or operational unbalance responses [13,34,35,38,39]. They also reveal a recurring limitation: several parameter combinations may reproduce similar dynamic responses. Increasing the number of adjustable parameters without increasing the independent information contained in the measurements can therefore reduce rather than improve the reliability of the estimated physical parameters.

### 5.2. Journal-Bearing and Rotor-Support Parameter Updating

Journal-bearing systems present additional difficulties because bearing forces and dynamic coefficients vary with rotational speed, load, lubricant properties, clearance, and journal equilibrium position. Consequently, uncertainty in the rotor structure should be reduced before bearing coefficients are interpreted as physically meaningful quantities.

Kang et al. applied a sequential model-updating procedure to a rotor–disk and journal-bearing system [14]. The structural properties of the rotor–disk model were first calibrated, after which the resulting model was used as the basis for journal-bearing dynamic-coefficient identification.

Sensitivity analysis was used to identify influential mass and stiffness variables, and particle swarm optimization was employed to estimate selected structural parameters. Damping information was incorporated using measured modal properties. After reducing uncertainty in the structural model, the journal-bearing coefficients were identified.

The importance of this sequential procedure lies in preventing the bearing parameters from compensating for errors elsewhere in the rotor model. If substantial uncertainty remains in shaft stiffness, disk properties, or support conditions, an inverse algorithm may reduce the overall residual by assigning unrealistic values to the bearing coefficients.

Chouksey et al. also investigated model updating for rotors supported on journal bearings [36]. Using a numerical rotor–journal-bearing model, the eccentricity ratio of the journal bearings and the shaft material damping coefficient were selected as physically meaningful updating parameters and identified using the inverse eigen-sensitivity method. This parameterization reduced the number of bearing-related updating variables by representing the journal-bearing coefficients through the eccentricity ratio rather than independently updating individual stiffness and damping coefficients.

For journal-bearing systems, however, coefficients identified under one operating condition should not automatically be assumed valid over the complete speed range. The direct and cross-coupled stiffness and damping terms represent a local linearization of fluid-film forces and can vary substantially with rotational speed and equilibrium state. Multi-speed validation is therefore particularly important [14,36,37].

Taherkhani and Ahmadian extended rotor-support updating into a stochastic framework [37]. Their study considered a complex turbo-compressor rotor–bearing–support system with speed-dependent hydrodynamic-bearing characteristics. Variance-based global sensitivity analysis was used to eliminate parameters with limited influence, followed by Bayesian updating to characterize uncertainty in the influential parameters.

The Bayesian formulation provides information that cannot be obtained from a single deterministic optimum. Instead of reporting only one parameter vector, posterior distributions describe the remaining uncertainty and correlation among estimated parameters. This is particularly relevant for rotating systems because bearing and support parameters are often only partially identifiable from limited measurements [37].

These studies indicate that support-parameter identification should preferably follow three stages: structural calibration, sensitivity or identifiability screening, and operating-condition-dependent bearing estimation. Without these stages, bearing parameters may act merely as numerical correction factors rather than physically meaningful quantities.

### 5.3. Operational-Response, Surrogate-Assisted, and Probabilistic FEMU

Traditional FEMU relies heavily on modal properties measured under stationary conditions. For rotating machinery, however, operational responses can provide information that is more directly related to speed-dependent rotor dynamics.

He et al. developed a rotor-model-updating method based on an adaptive Gaussian-process model using unbalance responses [49]. The approach established a computationally efficient mapping between rotor parameters and multi-speed operational responses, thereby reducing the number of repeated high-fidelity model evaluations required during parameter estimation.

The significance of operational-response-based FEMU is that it can incorporate response characteristics directly related to the intended rotor-dynamic prediction problem. Critical-speed location, resonance amplitude, phase evolution, and speed-dependent vibration contain information that may not be observable from stationary modal testing alone [34,35,39,49].

The operational-response perspective is also supported by Kang et al. [39], who demonstrated simultaneous estimation of bearing dynamic coefficients and residual unbalance using steady-state responses of a running rotor. Their results show that operational measurements can provide sufficient information for inverse estimation, while also highlighting the strong coupling that may exist among bearing and excitation parameters.

More recently, Wang et al. proposed an unbalance-response-based Bayesian model-updating framework for rotor systems using an adaptive hybrid-kernel Gaussian-process model [50]. The method combines measured unbalance responses, probabilistic parameter estimation, and surrogate-assisted computation within a single updating framework.

This development is important because it extends surrogate-assisted rotor FEMU beyond deterministic optimization. The Gaussian-process model reduces the computational burden of repeated numerical evaluation, whereas the Bayesian formulation describes posterior uncertainty in the estimated parameters [50].

Together, Han et al. [35], He et al. [49], and Wang et al. [50] illustrate a methodological progression from Kriging-assisted deterministic identification to adaptive Gaussian-process updating and surrogate-assisted Bayesian inference. In each case, the surrogate acts as a computational approximation of the parameter–response relationship, while the final quantities of interest remain physical parameters of the rotating system.

However, surrogate accuracy must be evaluated locally as well as globally. Rotor responses can change rapidly near resonance, critical speeds, mode interactions, and stability boundaries. A surrogate that is accurate over most of the parameter domain may still produce substantial error in the region that dominates parameter identification.

Accordingly, final parameter estimates obtained using a surrogate should be reintroduced into the original finite element model. The reconstructed high-fidelity model, rather than the surrogate alone, should then be evaluated using experimental responses or operating conditions not used directly during parameter estimation.

### 5.4. Active-Magnetic-Bearing Model Updating and AI-Assisted Inverse Estimation

Active-magnetic-bearing (AMB) systems provide an important application of FEMU because accurate rotor models are required not only for dynamic prediction but also for model-based controller design and stability assessment.

Wroblewski et al. presented an experimentally driven rotor-model updating and validation procedure for an active-magnetic-bearing-based high-speed machining spindle [54]. The study addressed discrepancies in the nominal finite element rotor representation using experimentally measured dynamic information and evaluated the resulting updated model.

Xu et al. subsequently proposed an experiment-based finite element model-updating method for an AMB rotor system [55]. Resonance information and mode-shape correlation based on the modal assurance criterion were incorporated into the updating procedure to improve agreement between the analytical rotor model and measured dynamic behavior.

More recently, Donati et al. developed an iterative finite element model-updating procedure for active-magnetic-bearing rotor systems used in practical turbomachinery applications [56]. The method adjusted the finite element model of a complex rotor using frequency measurements obtained from the actual rotor and subsequently used the updated representation for robust controller development [56].

The sequence of these studies demonstrates a progression from experimental rotor-model correlation toward direct integration of updated finite element models into downstream control-system design [54,55,56]. In actively controlled rotating machinery, model discrepancy can therefore affect not only predicted mechanical responses but also the performance and robustness of model-based control algorithms.

AI-based inverse methods provide a different route to computationally efficient parameter estimation. Noever-Castelos et al. applied a conditional invertible neural network to FEMU of a wind-turbine blade [19]. A parametric finite element database was used to learn the inverse relationship between modal information and uncertain structural parameters.

The use of an invertible architecture is relevant because the FEMU inverse problem may be non-unique. Rather than assuming that one measured response necessarily corresponds to one physical parameter combination, probabilistic or invertible models can represent multiple parameter combinations that remain compatible with the observed data [19].

However, AI-assisted updating introduces a different form of model dependency. The inverse estimator can only learn relationships represented in the simulation and experimental database used for training. Techniques demonstrated in fault-diagnosis and simulation-to-real studies [23,25,26,28], including noise-robust learning, synthetic-data generation, and domain adaptation, may help construct more representative databases for future AI-assisted FEMU; however, these studies do not themselves perform physical-parameter updating. AI-estimated parameters should therefore be regarded as candidate physical estimates. They should satisfy engineering bounds and subsequently be inserted into the original finite element model for validation against experimental responses not used during training or inverse estimation.

Thus, AMB and AI-assisted applications demonstrate two complementary developments of FEMU: extension toward experimentally updated models for actively controlled rotor systems and acceleration of inverse physical-parameter estimation. In both cases, the final criterion remains the predictive capability of the reconstructed physical model.

### 5.5. Nonlinear Rotor Model Updating and FEMU-Enabling Applications

Although nonlinear inverse model updating has begun to be demonstrated numerically for rotor–bearing systems [42], direct experimental FEMU remains most established for linear or moderately nonlinear rotor systems. Many studies involving nonlinear fluid-film behavior, rotor–stator rubbing, and coupled faults continue to focus primarily on forward simulation, uncertainty propagation, or comparison of simulated and experimental response characteristics.

These studies are relevant to FEMU because they identify the physical parameters, response quantities, and nonlinear mechanisms that future inverse procedures must consider. However, they should not be classified as direct FEMU when measured responses are not used to estimate physical parameters of the numerical model.

#### 5.5.1. Nonlinear Rotor–Bearing Model Updating and Bearing Instability

Zhu et al. [42] proposed a nonlinear rotor–bearing model-updating framework based on the multi-harmonic balance method and analytical response sensitivities. The method used nonlinear frequency-domain responses to iteratively update contact stiffness, bearing clearance, and eccentricity, and was further applied to a finite element rotor–bearing model. This study is particularly relevant because it explicitly closes the inverse parameter-estimation loop for strongly nonlinear bearing behavior, rather than using the nonlinear model only for forward response analysis. However, its validation was based on numerical cases, and laboratory experimental updating was not reported [42].

Garoli and de Castro investigated a nonlinear rotor–bearing system with uncertain bearing-related parameters using generalized polynomial chaos and stochastic collocation [17]. Their analysis quantified variability in nonlinear responses and examined rotational-speed regions associated with fluid-induced instability.

The study indicates that identification of nonlinear bearing behavior should not rely solely on synchronous vibration amplitude. Instability-onset speed, sub-synchronous frequency content, orbit characteristics, equilibrium position, and response variability may contain additional information regarding the underlying bearing parameters.

However, the experimental inverse loop was not completed by estimating the uncertain bearing parameters directly from measured responses. The study is therefore classified here as FEMU-enabling rather than direct FEMU.

#### 5.5.2. Nonlinear Rub-Impact and Multiple Response States

Fu et al. investigated a dual-rotor system with rub-impact under interval uncertainty [43]. The analysis considered nonlinear steady-state responses and the possibility of multiple valid solutions for the same parameter combination.

This behavior has an important implication for FEMU. If several stable response branches exist for the same physical model, matching only one vibration amplitude does not ensure that the correct parameter set or physical response branch has been identified.

Contact-related parameters such as clearance, contact stiffness, friction, and support properties may therefore require multi-response and multi-condition information. Parameter intervals or probability distributions may also be more meaningful than single deterministic estimates when experimental uncertainty and multiple response states are significant.

Because the study primarily addressed nonlinear forward response and uncertainty rather than experimentally driven inverse parameter estimation, it is treated as a FEMU-enabling study.

#### 5.5.3. Misalignment–Rubbing Interaction and Compound Faults

Tang et al. combined numerical and experimental analyses of a rotor–bearing system subjected to coupled misalignment and rubbing [45]. The resulting behavior demonstrated that simultaneous faults cannot necessarily be represented as a simple superposition of independently modeled fault responses.

This finding is directly relevant to future FEMU. Misalignment may redistribute bearing loads, alter rotor equilibrium position, modify effective clearance, and thereby influence the development of rotor–stator rubbing. Estimating misalignment and rubbing parameters independently can therefore produce incorrect physical interpretations when the two mechanisms interact.

The study provides experimentally supported response characteristics for coupled-fault modeling but does not close the inverse loop by identifying misalignment, clearance, contact stiffness, or friction directly from experimental data. It is therefore classified as a FEMU-enabling application.

The nonlinear studies reviewed above show that the next stage of rotating-machinery FEMU will require more than conventional residual minimization. Nonlinear inverse procedures must account for response-branch dependence, coupled parameters, model-form uncertainty, and operating-history effects [17,43,44,45,46].

### 5.6. Comparative Analysis of Representative Applications

To complement the conceptual relationships summarized in Figure 4 with case-based evidence from the reviewed literature, Table 2 compares representative direct FEMU studies and FEMU-enabling applications according to the system considered, parameters of interest, data and updating method, validation level, principal contribution, and main limitation.

The comparison indicates that the strongest direct evidence for rotating-machinery FEMU currently concerns rotor–bearing calibration, bearing and support identification, operational-response-based parameter estimation, probabilistic rotor updating, and experimentally correlated AMB rotor models [13,14,34,35,36,37,38,39,49,50,54,55,56].

A second observation is that successful applications commonly reduce or structure the inverse problem before final estimation. Sensitivity analysis, physical parameter constraints, sequential calibration, surrogate modeling, probabilistic inference, and coupled-state estimation are used to reduce ambiguity among weakly observable variables [14,35,37,39,49,50].

A third observation concerns validation. Reduction of the residual used during parameter estimation is necessary but insufficient. Stronger FEMU studies evaluate whether the updated model reproduces additional modal quantities, frequency response functions, unbalance responses, operating conditions, or downstream model-based behavior that were not represented solely by the calibration objective [13,39,54,55,56].

The recent studies by Kang et al. [39], Wang et al. [50], and Donati et al. [56] also indicate three emerging directions: operational estimation of coupled rotor parameters, probabilistic updating accelerated by surrogate models, and integration of experimentally updated rotor models with model-based control.

In contrast, nonlinear bearing, rub-impact, and compound-fault studies demonstrate that the physical mechanisms required for future FEMU are becoming increasingly sophisticated, while experimentally validated inverse estimation remains comparatively limited. This gap is particularly important for clearance, friction, contact stiffness, nonlinear fluid-film parameters, and interacting fault parameters [17,43,44,45,46].

The reviewed applications therefore suggest three principal priorities for rotating-machinery FEMU: multi-condition physical-parameter identification, explicit uncertainty treatment, and experimentally grounded predictive validation. Computational acceleration through surrogate or AI methods can support these objectives, but it should not replace reconstruction and evaluation of the updated physical model.

## 6. FEMU-Enabled Condition Monitoring, Digital Twins, and Future Research Directions

The reviewed literature indicates that FEMU is evolving from an offline calibration procedure toward broader applications involving operational parameter estimation, condition assessment, prognostics, and synchronization of physics-based models with operating machinery [13,14,29,30,31,32,33,34,35,36,37,38,39,49,50]. Experimental evidence is relatively well established for rotor and bearing parameter identification and operational-response-based model updating, whereas long-term prognostics and continuously synchronized digital-twin applications remain less extensively validated [13,14,34,35,36,37,38,39,49,50].

To distinguish established findings from forward-looking concepts, Section 6.1 and Section 6.2 summarize capabilities supported by the reviewed literature. Section 6.3, Section 6.4, Section 6.5 and Section 6.6 then present the authors’ proposed lifecycle-oriented framework, validation and governance requirements, research priorities, and development roadmap derived from the gaps identified in the preceding sections.

### 6.1. Reviewed Evidence: FEMU for Condition Monitoring and Prognostics

Finite element model updating can extend conventional vibration monitoring by relating persistent model–measurement discrepancies to changes in physically interpretable parameters. Rather than treating variations in vibration amplitude, spectral components, phase, or shaft orbit only as signal-based indicators, FEMU can evaluate whether these changes are consistent with variations in bearing stiffness, damping, support flexibility, shaft stiffness, unbalance, clearance, or other physical model parameters [13,14,34,35,36,37,38,39,49,50].

This capability does not imply that every estimated parameter change represents physical degradation. Rotor responses also vary with rotational speed, load, temperature, lubrication condition, alignment, bearing state, and measurement environment [14,15,34,35,36,37,38,39,48]. Accordingly, FEMU-based condition assessment should interpret estimated parameter changes together with the corresponding operating conditions.

Repeated updating may provide a time history of estimated physical parameters and can potentially support degradation tracking when the estimated changes are sufficiently observable and consistent with the operating state [29,30,31]. When a physically interpretable parameter exhibits a persistent trend, a temporal or data-driven model may be used to project its future evolution, while the finite element model can be used to evaluate the corresponding dynamic consequences, including changes in vibration response, critical speed, stability margin, or rotor–stator clearance [29,30,31].

This separation is attractive because degradation evolution and dynamic-response prediction do not necessarily need to be represented by the same model. A statistical or data-driven temporal model may describe the evolution of an estimated physical parameter, whereas the finite element model preserves the mechanical relationship between the parameter state and the resulting rotor response.

Depending on the available degradation history, the temporal evolution of an updated physical parameter may be represented using a linear or exponential trend model, a stochastic process, a state-space formulation, or a data-driven temporal predictor. RUL estimation additionally requires a physically defined failure criterion, which may be specified directly as a limiting physical parameter value, such as allowable bearing stiffness or clearance, or indirectly through a finite-element-predicted response threshold, such as a minimum stability margin, critical rotor–stator clearance, or permissible vibration level. Because uncertainty exists in the measurements, updated parameters, degradation-model parameters, future operating conditions, and finite element response prediction, these uncertainties should be propagated through the forecasting process so that RUL is expressed as a distribution or confidence interval rather than only as a deterministic value.

Calibrated finite element models can also support synthetic-data generation for fault diagnosis. Simulation-generated data are increasingly used in machine-learning-based condition monitoring, transfer learning, and hybrid physics–AI diagnosis [20,21,22,23,24,25,26,27,28]. A FEMU-calibrated baseline model may improve the physical consistency of such datasets by reducing discrepancies between the nominal numerical representation and the measured healthy machine before fault scenarios are introduced [13,14,25,26].

However, calibration of the healthy model does not by itself validate the subsequent fault representation. Accurate reproduction of the baseline rotor response, for example, does not demonstrate that an assumed rubbing, misalignment, nonlinear bearing, or fluid-induced instability model adequately represents the corresponding physical fault mechanism [17,40,41,42,43,44,45,46].

**Figure 5:**
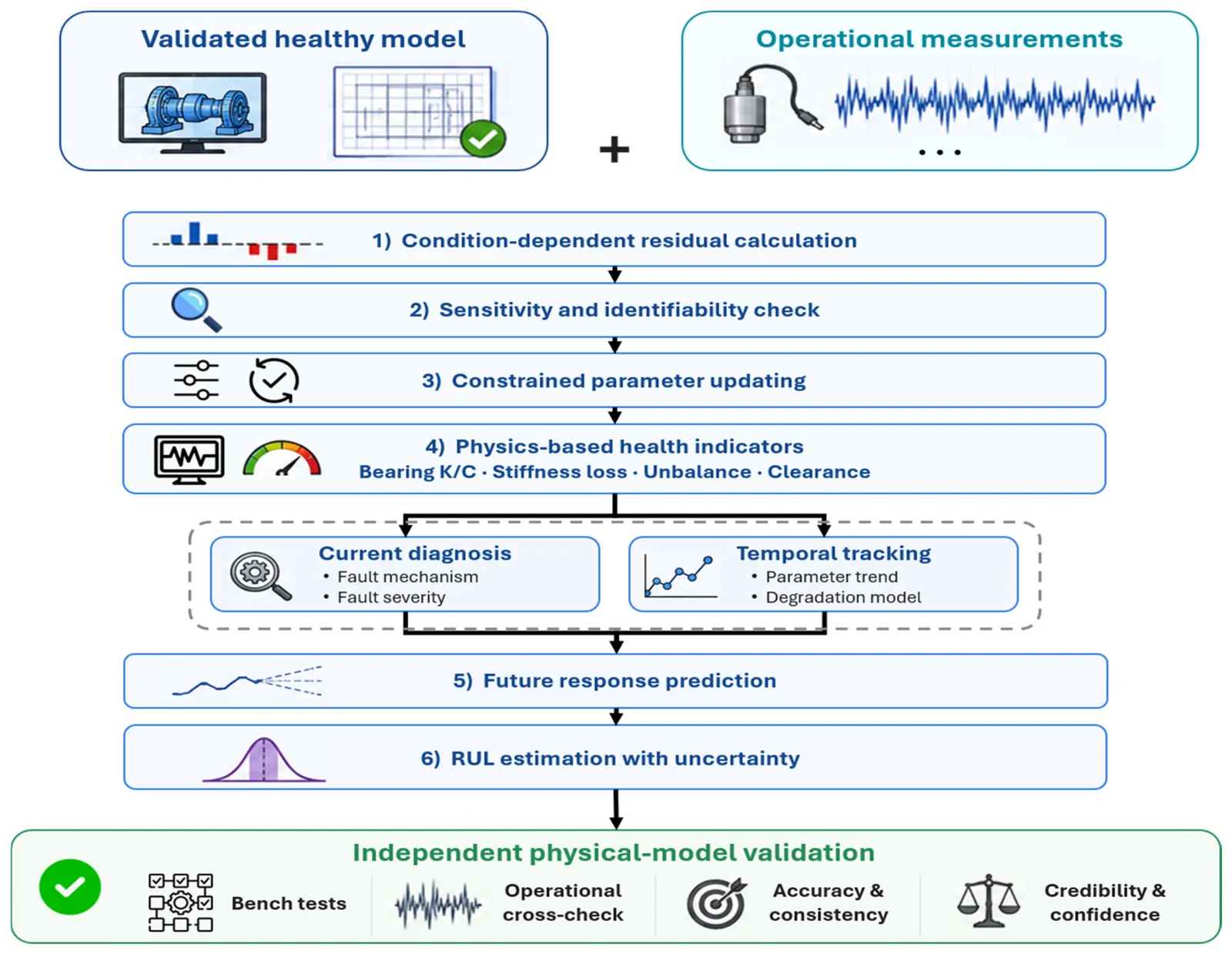
Conceptual FEMU-based framework for condition assessment, fault-severity estimation, degradation tracking, and prognostics; specific degradation models, failure criteria, and uncertainty-propagation requirements are discussed in Section 6.1.

Based on the reviewed evidence and the relationships identified above, Figure 5 presents the authors’ synthesis of a FEMU-based pathway from model–measurement comparison to condition assessment, degradation tracking, and prognostic evaluation.

As illustrated in Figure 5, estimated physical parameters may serve as condition indicators or fault-severity variables and, when sufficient temporal information is available, as inputs for degradation forecasting. Nevertheless, the reviewed evidence for long-term FEMU-based prognostics remains limited, particularly because datasets containing repeated vibration measurements together with independently observed physical degradation are scarce [30,31]. Consequently, many FEMU–prognostic applications should currently be regarded as methodological frameworks rather than fully demonstrated lifecycle solutions.

### 6.2. Reviewed Evidence: FEMU as a Digital-Twin Synchronization Mechanism

A digital twin extends the concept of model updating from one-time calibration toward repeated synchronization between a physical machine and its virtual representation [29,30,31,32,33]. Within this context, FEMU provides a physically interpretable mechanism for correcting a numerical model when persistent discrepancies arise between predicted and measured responses.

The connection of sensor data to a finite element model alone does not constitute reliable synchronization. The essential function is an inverse process that determines whether observed discrepancies can be explained by changes in identifiable physical parameters and whether modification of the numerical model is justified [7,12,29,30].

This issue is particularly important for rotating machinery because measured responses vary strongly with rotational speed, load, thermal state, alignment, bearing condition, and support behavior [14,15,30,34,35,36,37,38,39]. A synchronized virtual representation should therefore distinguish normal operating-condition dependence from physical changes that require modification of the model.

Wang et al. demonstrated the use of sensitivity-based model updating and experimental rotor measurements to quantify and localize progressive unbalance [29]. The significance of this application lies in repeated correction of physical model parameters rather than in online visualization of vibration measurements alone. Such studies provide evidence that FEMU can function as a model-synchronization mechanism when the changing physical quantity is observable from the available measurements.

Recent digital-twin and FEMU studies also emphasize surrogate models, reduced-order representations, and data-driven estimators to reduce the computational burden associated with repeated physical-model evaluation [30,31,32,33,35,49,50]. These approaches are particularly relevant when direct execution of a high-fidelity finite element model cannot satisfy the required update interval.

However, rapid surrogate or neural-network inference alone does not establish real-time FEMU, because practical real-time capability depends on the end-to-end synchronization latency of the complete updating process, as further discussed in Section 6.3.

The reviewed evidence therefore supports FEMU as a promising physical synchronization mechanism for rotating-machinery digital twins, while continuously validated and fully autonomous lifecycle implementations remain an emerging research area [30,31,32,33].

### 6.3. Authors’ Proposed Lifecycle-Oriented FEMU–Digital-Twin Framework

Based on the reviewed evidence on rotating-system FEMU, computationally accelerated updating, and digital-twin synchronization [29,30,31,32,33,34,35,49,50], the authors propose a lifecycle-oriented architecture in which FEMU connects a high-fidelity physical model with operational measurements, condition assessment, and future-response prediction.

The proposed architecture consists of two interacting levels: an offline high-fidelity layer and an online synchronization layer.

The offline layer comprises:

1. development of the baseline finite element rotor model;
2. experimental calibration of structural, bearing, and support properties;
3. sensitivity and identifiability analysis;
4. selection of parameters suitable for repeated updating;
5. definition of relevant parameter and operating domains;
6. development and verification of reduced-order or surrogate models when computational acceleration is required.

The objective of the offline stage is to establish a physically credible reference model and to define the parameter and operating domains within which subsequent updating can be performed.

The online layer comprises:

1. acquisition of vibration and operating-condition measurements;
2. preprocessing into response quantities directly comparable with model outputs;
3. evaluation of model–measurement residuals;
4. estimation of selected physical parameters or system states;
5. reconstruction of the synchronized numerical model;
6. use of the synchronized model for condition assessment or future-response prediction.

**Figure 6:**
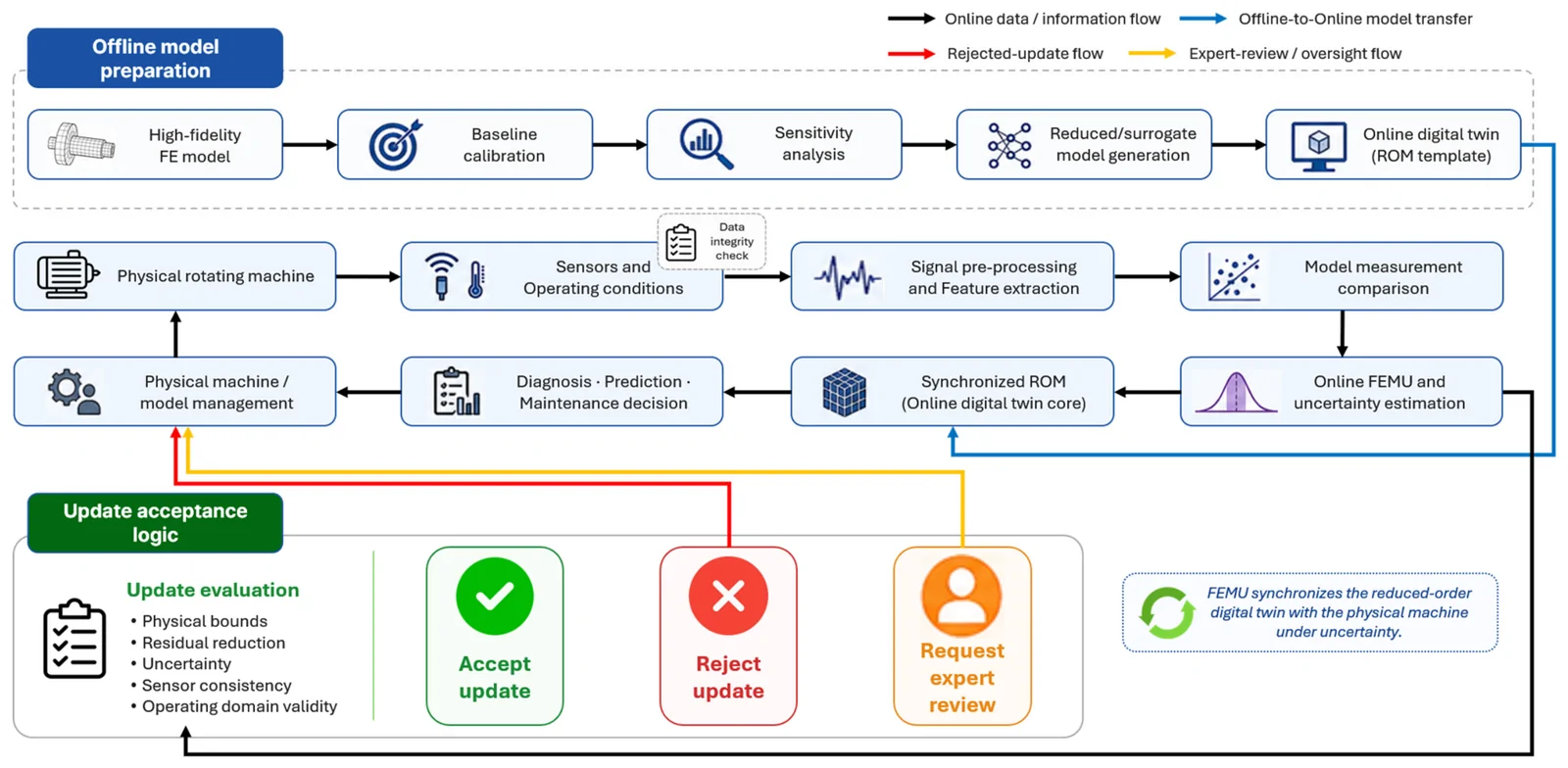
Proposed FEMU-enabled digital twin architecture and synchronization loop for rotating machinery.

Figure 6 summarizes the authors’ proposed offline–online architecture.

As shown in Figure 6, routine online estimation does not necessarily require direct execution of the original high-fidelity finite element model at every update cycle. A reduced-order model, surrogate model, or rapid inverse estimator may be used for computationally efficient estimation, while the high-fidelity model remains the physical reference for reconstruction and periodic verification. Practical implementation should therefore consider not only estimation accuracy but also available computing resources, data-acquisition and preprocessing time, end-to-end update latency, data-transfer and storage requirements, and the required synchronization frequency. The selection between high-fidelity, reduced-order, surrogate, and AI-assisted implementations should also account for sensor infrastructure, model maintenance, periodic revalidation, and the associated operation and maintenance cost.

Measurement selection should reflect the physical parameters being estimated. Shaft displacement and phase may provide information related to unbalance, whirl, and clearance, whereas casing acceleration may be more suitable for global modal and forced-response characteristics. Rotational speed, load, and temperature should be included whenever they influence the modeled responses or estimated parameters.

Within the proposed framework, condition monitoring and prognostics are applications of the synchronized physical model rather than independent services detached from the FEMU loop. The same estimated parameter history can therefore support current-state assessment, fault-severity estimation, and prediction of future dynamic behavior.

### 6.4. Validation, Update Governance, and Domain Control

The validation and governance requirements proposed here synthesize recurring issues identified in FEMU concerning parameter identifiability, uncertainty, changing operating conditions, model-form inadequacy, and data-domain dependence [7,10,11,12,15,19,23,26,28,37,47,48,51].

A proposed model update should be evaluated at three levels.

- Parameter level: the estimated quantities should remain consistent with physically admissible ranges, the known machine configuration, and plausible rates of change.
- Response level: the reconstructed model should reproduce measured behavior beyond the specific response quantities used directly for parameter estimation.
- Model level: persistent residual patterns should be examined to determine whether they can reasonably be explained by parameter variation or instead indicate inadequacy of the assumed physical model.

Such systematic discrepancies may indicate model-form inadequacy rather than actual parameter variation, as discussed in Section 3.6. Residual reduction alone is therefore insufficient evidence of physically successful updating and should be evaluated together with parameter plausibility and predictive validation.

Update governance should include an accept, reject, or review decision. An estimated update may be accepted when the parameter change is physically interpretable, sufficiently observable, and supported by the available response information. It should be rejected or referred for further review when parameter estimates become unstable or strongly non-unique, different measurements provide conflicting evidence, or the measured behavior cannot be represented adequately by the available model class.

Domain control is particularly important for surrogate- and AI-assisted estimation because predictions may become unreliable outside the parameter or operating ranges represented during model development [19,23,26,28,35,49,50]. If a measured condition lies outside the validated parameter, operating, or fault domain, the estimation system should identify the case as outside its validated domain rather than return an apparently precise but unsupported parameter estimate.

For lifecycle applications, each accepted update should also retain traceable information regarding operating condition, sensor configuration, numerical-model version, estimated uncertainty, and maintenance state. Such traceability is necessary to distinguish genuine physical changes from apparent changes caused by sensor replacement, preprocessing modifications, maintenance actions, or model revision.

### 6.5. Remaining Research Gaps and Future Directions

The reviewed literature identifies five research areas that are particularly important for advancing rotating-machinery FEMU.

#### 6.5.1. Nonlinear and Model-Form-Aware FEMU

As summarized in Section 5.5, experimentally validated inverse FEMU for nonlinear rotor behavior remains less mature than forward nonlinear modeling, particularly for fluid-film, clearance, friction, rotor–stator contact, and interacting fault parameters [17,40,41,42,43,44,45,46].

Future FEMU should therefore target parameters associated directly with the relevant nonlinear force mechanisms rather than representing all nonlinear effects using equivalent constant stiffness or damping. Candidate parameters may include bearing clearance, lubricant-dependent coefficients, contact stiffness, friction parameters, misalignment, and state-dependent support properties.

Measurements should also extend beyond stationary natural frequencies and synchronous amplitudes. Shaft orbits, sub-synchronous components, run-up and coast-down responses, instability-onset speeds, hysteresis, jump behavior, and bifurcation characteristics may provide information required to distinguish nonlinear parameter changes from inadequacy of the selected model form.

The central research problem is therefore not only the development of more detailed forward models but also the formulation of inverse procedures capable of determining whether the observed discrepancy arises from uncertain parameters or from missing physical mechanisms.

#### 6.5.2. Identifiability and Measurement Design

Parameter estimation should be preceded by explicit identifiability analysis. Shaft stiffness, bearing stiffness, support flexibility, damping, excitation, and fault-related parameters can produce correlated effects on measured vibration responses [7,12,34,35,36,37,38,39,51].

Future studies should therefore design the candidate parameter set, sensor configuration, response quantities, rotational-speed points, and excitation conditions jointly rather than treating measurement design and parameter selection as independent tasks.

Multi-speed and multi-response measurements are particularly valuable because the sensitivity of different parameters can vary substantially across operating conditions [14,34,35,37,38,39,49,50]. Such measurements may provide independent information that cannot be obtained from a single resonance, frequency, or operating point.

#### 6.5.3. Computationally Efficient and Physics-Consistent AI

Surrogate models, Gaussian-process models, reduced-order representations, and neural-network inverse estimators can substantially reduce the computational cost of repeated FEMU [9,19,35,49,50]. Future development should focus not only on inference speed but also on the reliability of the complete estimation and synchronization process.

Training databases should represent relevant variations in rotational speed, load, temperature, bearing properties, support flexibility, assembly conditions, fault severity, sensor behavior, and machine-to-machine variability [19,23,25,26,28].

Probabilistic or invertible architectures may be particularly useful when the inverse relationship is non-unique [19,50]. Nevertheless, the performance of AI-assisted FEMU should ultimately be evaluated through physical parameter recovery and predictive rotor response rather than through regression accuracy alone.

#### 6.5.4. Lifecycle Updating and Compound Faults

Industrial rotating machines experience balancing, maintenance, component replacement, sensor changes, temporary operating variations, and gradual degradation throughout their service life [29,30,31,32,33]. Lifecycle-oriented FEMU should therefore distinguish persistent degradation from discrete changes in machine configuration or measurement conditions.

Compound faults provide an additional challenge because different physical mechanisms may interact. Misalignment can alter bearing load distribution and rotor equilibrium position, while changes in unbalance or support conditions can influence the onset of rotor–stator contact [43,44,45,46]. Future FEMU should therefore consider competing physical models and interacting fault parameters rather than assuming that compound-fault responses are simple superpositions of individual faults.

Parameter histories should also be interpreted together with maintenance records and operating conditions. Following balancing, bearing replacement, alignment correction, or other maintenance actions, some previously estimated parameters may remain applicable, whereas others may require reinitialization.

#### 6.5.5. Standardized Benchmarking

Comparison among existing FEMU studies remains difficult because rotor configurations, uncertain parameters, measurement quantities, operating ranges, and validation procedures differ substantially.

Future benchmark datasets should include raw measurements acquired over multiple rotational speeds and load conditions, controlled healthy and fault or parameter-change conditions with different severity levels, and repeated measurements or predefined noise levels where possible. Independently known or measured reference parameters and controlled physical modifications should be provided as ground truth for evaluating parameter-recovery accuracy. The associated metadata should include rotational speed, load, temperature, sensor type, location and orientation, sampling frequency, calibration information, excitation conditions, and machine state.

At minimum, future FEMU studies should report the initial model discrepancy, candidate parameter set, sensitivity or parameter-correlation information, clearly separated calibration, validation, and independent test data, estimated parameter values, uncertainty, computational cost, and rejected or non-convergent updating cases.

Such benchmark datasets would enable more direct comparison of sensitivity-based, optimization-based, Bayesian, surrogate-assisted, and AI-driven FEMU under equivalent physical conditions.

### 6.6. Authors’ Proposed Research Roadmap

Based on the methodological progression and unresolved limitations identified in the reviewed literature, the authors propose a four-stage roadmap toward lifecycle-oriented FEMU-enabled digital twins. These stages represent interacting capability levels rather than a strictly sequential development pathway. In practical engineering applications, nonlinear modeling, uncertainty treatment, computational acceleration, and lifecycle synchronization may develop concurrently or overlap according to application requirements, available data, and computational resources.

Stage 1: Reliable Offline FEMU

The first stage establishes a physically meaningful baseline model through experimental calibration, parameter screening, sensitivity and identifiability analysis, and prediction of responses beyond those used directly for updating.

Stage 2: Nonlinear and Uncertainty-Aware FEMU

The second stage extends FEMU toward nonlinear bearing behavior, rotor–stator interaction, fault-related parameters, and explicit uncertainty representation. Model-form comparison becomes increasingly important as the number and complexity of possible physical mechanisms increase.

Stage 3: Computationally Efficient and AI-Assisted FEMU

The third stage introduces reduced-order models, adaptive surrogates, and physics-consistent inverse learning to enable repeated or near-real-time parameter estimation while retaining connection with the high-fidelity physical model.

Stage 4: Lifecycle FEMU-Enabled Digital Twin

The final stage integrates repeated model synchronization with parameter histories, multimodal measurements, maintenance information, compound-fault modeling, uncertainty tracking, and governed update decisions.

Table 3 summarizes the proposed four-stage research roadmap.

**Figure 7:**
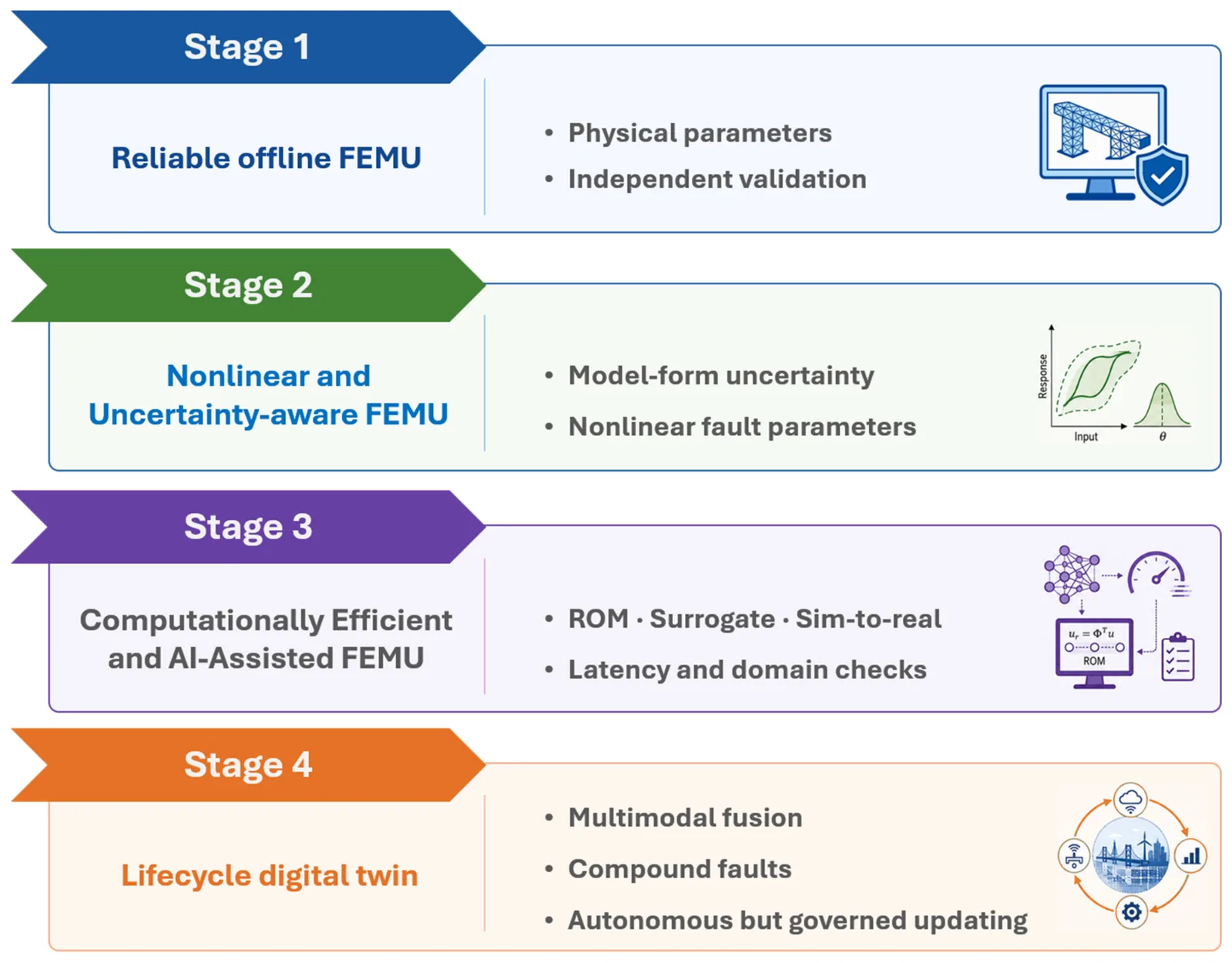
Proposed research roadmap toward lifecycle-oriented FEMU-enabled digital twins.

Figure 7 summarizes the proposed capability-oriented roadmap toward increasing model fidelity, computational efficiency, uncertainty awareness, and controlled autonomy.

The proposed roadmap should not be interpreted as evidence that fully autonomous FEMU-enabled digital twins have already been demonstrated for rotating machinery. Rather, it represents the authors’ synthesis of the developments required to connect the experimentally established capabilities of conventional FEMU with emerging computational, AI, and digital-twin technologies.

## 7. Conclusions

This review examined finite element model updating for rotating machinery from its methodological foundations to its emerging roles in condition monitoring and digital-twin synchronization. Particular attention was given to the characteristics that distinguish rotating machinery from stationary structures, including speed-dependent dynamics, bearing and support uncertainty, gyroscopic effects, nonlinear forces, limited measurement availability, and parameter correlation. The reviewed studies were further distinguished between direct FEMU applications, in which experimental measurements are used to estimate physical model parameters, and FEMU-enabling studies that provide nonlinear models, uncertainty-analysis methods, surrogate techniques, or AI-based inverse tools.

The reviewed evidence indicates that FEMU is currently most mature for rotor–bearing model calibration, structural and support parameter estimation, bearing-coefficient identification, and operational-response-based updating. Sensitivity-based and optimization-based methods remain central to physically interpretable parameter estimation, while Bayesian approaches provide explicit uncertainty representation and surrogate or AI-assisted methods offer computational acceleration. These methods should therefore be regarded as complementary rather than competing approaches, with their suitability depending on parameter dimensionality, response nonlinearity, measurement quality, uncertainty, and computational requirements.

A major gap remains between advanced nonlinear forward rotor models and experimentally validated inverse parameter identification. Further progress requires improved identifiability, multi-condition measurement design, uncertainty-aware estimation, and experimentally supported model-form assessment.

The literature also indicates a transition from one-time offline calibration toward repeated synchronization of physics-based models with operating machinery. However, long-term prognostic and lifecycle digital-twin implementations remain less mature than conventional offline FEMU. The proposed architecture and roadmap therefore synthesize the developments required to connect established FEMU capabilities with emerging computational and digital technologies.

Overall, the future significance of rotating-machinery FEMU lies not in achieving the smallest calibration residual, but in constructing models whose estimated parameters retain physical meaning and whose predictions remain credible as machine condition and operating state change. Advances in nonlinear modeling, uncertainty quantification, computational acceleration, and AI can substantially extend this capability, provided that they remain integrated with physically interpretable finite element models and experimentally grounded evaluation. In this form, FEMU can evolve from an offline calibration technique into a core enabling methodology for physics-based condition assessment and lifecycle-oriented digital representations of rotating machinery.

## Figures and Tables

**Table 1:** Comparison of principal FEMU approaches for rotating machinery.

Method	Updating Target	Main Input	Main Advantage	Main Limitation	Suitable Rotating-Machinery Use
Direct matrix correction	Assembled system matrices	Modal data	Rapid numerical correlation	Weak physical interpretation	Initial model correlation and discrepancy localization
Sensitivity-based	Physical parameters	Frequencies, modes, FRFs, operational responses	Efficient and physically interpretable	Local linearity and ill-conditioning	Moderate and identifiable rotor–bearing parameter corrections
Optimization-based	Physical or fault-related parameters	Modal and operational responses	Flexible objective functions and parameter constraints	Computational cost and non-uniqueness	Nonlinear or multi-response parameter estimation
Surrogate-assisted	Physical parameters through an approximate model	Parametric numerical database	Rapid repeated evaluation	Training-domain dependence and approximation error	Computationally expensive or multi-speed FEMU
Bayesian/ probabilistic	Parameter distributions	Noisy or incomplete measurements	Explicit uncertainty quantification	Expensive posterior inference	Uncertain bearing and support parameter estimation
AI-driven inverse	Parameters or parameter distributions	Simulation and experimental database	Rapid inverse estimation	Generalization, identifiability, and simulation-to-experiment discrepancy	Repeated estimation within a validated domain
Hybrid	Combined physical parameters and uncertainty	Multiple model and response sources	Balance of interpretability, robustness, and efficiency	Greater implementation complexity	High-fidelity calibration and repeated operational updating

**Table 2:** Comparative summary of representative direct FEMU and FEMU-enabling applications for rotating systems.

Study and System	Updated or Uncertain Parameters	Data and Method	Classification and Principal Contribution	Validation Level and Main Limitation
Chouksey et al. [13]—ball-bearing-supported rotor	Bearing stiffness and damping; shaft material damping	Modal data and FRFs; inverse eigen-sensitivity	**Direct FEMU**; physical rotor–bearing calibration followed by forced-response prediction and balancing	Laboratory experimental validation; demonstrated on a single ball-bearing-supported rotor configuration
Kim et al. [34]—rotor–bearing system	Bearing parameters and disk-unbalance quantities	Measured unbalance responses; hybrid evolutionary optimization	**Direct FEMU**; experimental bearing-parameter and unbalance identification	Numerical and laboratory experimental validation; focused on bearing parameters and disk unbalance in a specific rotor-bearing configuration
Han et al. [35]—rotor–bearing system	Bearing parameters	FEM database and measured responses; Kriging surrogate and differential evolution	**Direct FEMU**; surrogate-assisted experimental parameter identification	Numerical and laboratory experimental validation; surrogate accuracy depends on the sampled parameter domain and training design
Chouksey et al. [36]—journal-bearing rotor	Rotor and journal-bearing-related parameters	Numerical modal properties; inverse eigen-sensitivity method	**Direct FEMU**; journal-bearing-supported rotor calibration	Numerical validation; experimental validation was not reported, and the bearing model was based on the short-bearing approximation
Kang et al. [14]—rotor–disk/journal-bearing system	Structural properties, damping, and journal-bearing coefficients	Modal data; sensitivity analysis and particle swarm optimization	**Direct FEMU**; sequential structural calibration and bearing-coefficient identification	Laboratory experimental validation; demonstrated on a rotor-disc test rig with a specific structural and bearing configuration
Jha et al. [38]—rotor system	Rotor-system physical parameters	Measured and calculated dynamic responses; nonlinear least squares	**Direct FEMU**; optimization-based physical-parameter updating	Laboratory experimental validation at multiple rotational speeds; focused mainly on deterministic bearing-parameter updating without explicit uncertainty quantification
Kang et al. [39]—double-disk rotor–bearing system	Bearing dynamic coefficients and residual unbalance	Steady-state unbalance responses; dual augmented Kalman filter	**Direct parameter identification supporting FEMU**; simultaneous operational estimation under multiple speeds and measurement conditions	Multi-speed experimental validation; demonstrated on a double-disk rotor-bearing system, with broader machine-level applicability yet to be established
Taherkhani and Ahmadian [37]—turbo-compressor rotor–bearing–support system	Uncertain rotor/support parameters	Global sensitivity analysis and Bayesian inference	**Stochastic FEMU**; posterior parameter estimation and uncertainty quantification	Measured-response stochastic validation; Bayesian inference involves relatively high computational cost and depends on the assumed prior and model form
He et al. [49]—rotor system	Rotor-system parameters	Multi-speed unbalance responses; adaptive Gaussian-process model	**Surrogate-assisted FEMU**; computational acceleration of operational-response updating	Numerical and laboratory experimental validation at multiple speeds; surrogate reliability remains dependent on the sampled parameter and operating domain
Wang et al. [50]—rotor system	Uncertain rotor-model parameters	Measured unbalance responses; Bayesian updating with adaptive hybrid-kernel Gaussian process	**Bayesian surrogate-assisted FEMU**; probabilistic operational-response-based model updating	Numerical and laboratory case-study validation; performance depends on surrogate training coverage and Bayesian sampling
Wroblewski et al. [54]—AMB high-speed spindle	Rotor-model parameters	Experimental dynamic measurements and FE correlation	**Direct FEMU**; experimentally driven rotor-model updating and validation	Laboratory experimental validation; demonstrated for an AMB-supported high-speed spindle with system-specific modal characteristics
Xu et al. [55]—AMB rotor	Rotor-model physical properties	Resonance and mode-shape information; MAC-based updating	**Direct FEMU**; experiment-based updating of an AMB rotor	Laboratory experimental validation using frequency-response data; demonstrated for one AMB rotor and selected model parameters
Donati et al. [56]—AMB rotor system	Complex rotor FE-model parameters	Actual-rotor frequency measurements; iterative optimization-based updating	**Direct FEMU**; updated rotor model used for robust controller development	Experimental validation using an actual AMB rotor system; focused on controller-oriented model updating based primarily on frequency-domain information
Noever-Castelos et al. [19]—wind-turbine blade	Structural and material parameters	Modal-response database; conditional invertible neural network	**AI-assisted FEMU**; rapid inverse estimation and representation of non-unique solutions	Simulation-based validation using FE-generated test samples; validation with real measured data was not performed
Garoli and de Castro [17]—nonlinear journal-bearing rotor	Bearing and operating uncertainties	Nonlinear/stochastic response analysis	**FEMU-enabling**; uncertainty characterization near fluid-induced instability	Numerical nonlinear and stochastic validation; focused on forward uncertainty analysis rather than experimentally validated inverse FEMU
Fu et al. [43]—dual-rotor rub-impact system	Contact and uncertain physical parameters	Nonlinear response and interval analysis	**FEMU-enabling**; uncertainty and multiple nonlinear response branches	Numerical validation; focused on forward uncertainty propagation of nonlinear rub-impact responses rather than inverse parameter updating
Tang et al. [45]—rotor–bearing system with misalignment and rubbing	Misalignment- and contact-related quantities	Numerical and experimental vibration responses	**FEMU-enabling**; interaction between coupled fault mechanisms	Numerical and laboratory experimental validation; focused on forward analysis of coupled misalignment–rubbing behavior rather than inverse FEMU
Zhu et al. [42]—nonlinear rotor–bearing system	Bearing contact stiffness, clearance, and eccentricity	Nonlinear frequency-domain responses; multi-harmonic balance and analytical inverse sensitivity	**Nonlinear model updating**; inverse identification of nonlinear bearing parameters	Numerical validation using bearing and FE rotor-bearing cases; laboratory experimental validation was not reported

**Table 3:** Research roadmap for rotating-machinery FEMU.

Capability Level	Principal Objective	Required Developments	Minimum Evidence
Reliable offline FEMU	Physically meaningful baseline calibration	Sensitivity and identifiability analysis, parameter screening, constrained updating	Prediction of additional responses or operating conditions beyond those used for calibration
Nonlinear and uncertainty-aware FEMU	Identification of nonlinear, fault, contact, and instability parameters	Nonlinear force models, uncertainty quantification, and model-form comparison	Experimental characterization of nonlinear responses and parameter uncertainty
Computationally efficient and AI-assisted FEMU	Repeated or near-real-time synchronization	Reduced-order models, adaptive surrogates, and rapid inverse estimation	Approximation accuracy, end-to-end latency, and parameter-tracking performance
Lifecycle FEMU-enabled digital twin	Long-term synchronization and decision support	Multimodal data, parameter histories, maintenance integration, compound-fault models, and update governance	Long-term experimental or industrial demonstration

## Data Availability

No new data were created or analyzed in this study. Data sharing is not applicable to this article.
